# Impact of Thermoelectric Power Plant Operations and Water Use Reporting Methods on Thermoelectric Power Plant Water Use

**DOI:** 10.1021/acs.est.4c02024

**Published:** 2025-02-27

**Authors:** Eric Sjöstedt, Richard Rushforth, Vincent Tidwell, Melissa Harris, Ryan McManamay, Landon Marston

**Affiliations:** † School of Informatics, Computing, And Cyber Systems, Northern Arizona University, Flagstaff, Arizona 86011, United States; ‡ Pacific Northwest National Laboratories, Richland, Washington 99354, United States; § U.S. Geological Survey, Lower Mississippi-Gulf Water Science Center, Nashville, Tennessee 37211, United States; ∥ Department of Environmental Science, Baylor University, Waco, Texas 76706, United States; ⊥ Department of Civil & Environmental Engineering, Virginia Tech, Blacksburg, Virginia 24061, United States

**Keywords:** thermoelectric power plants, power-cooling ratio, energy-water nexus, water withdrawal and consumption

## Abstract

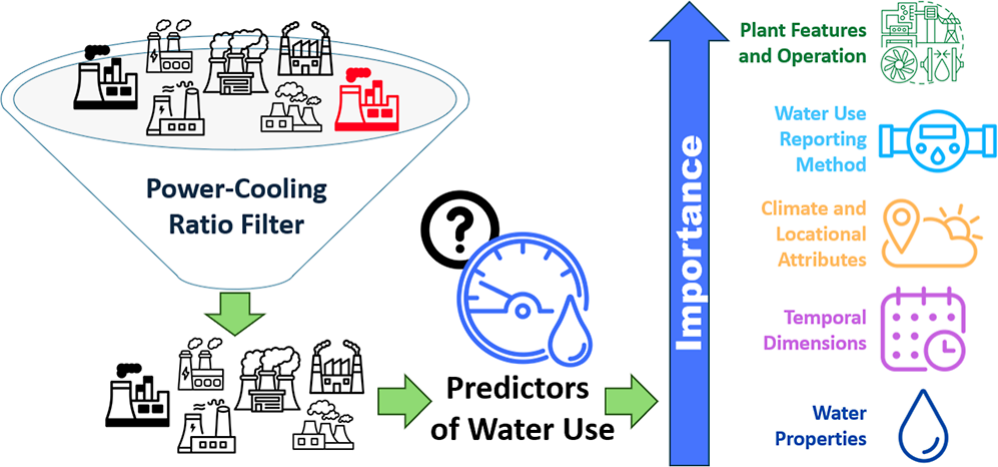

Thermoelectric power generation accounts for over 41% of total U.S. freshwater withdrawals, making understanding the determinants of power plants’ water withdrawals (WW) and consumption (WC) critical for reducing the sector’s reliance on increasingly scarce water resources. However, reported data inconsistencies and incomplete analysis of potential determinants of thermoelectric water use hinder such understanding. We address these challenges by introducing a novel data filtering method and a more complete assessment of water use determinants. First, we applied a power-cooling ratio as an operations-based data filter that removed operationally implausible records while retaining more original data, outperforming previous statistical filtering methods. Second, we found that different water use reporting methods (WURMs) provided statistically significantly different WW and WC values, revealing the importance of this previously unrecognized feature in reported water use records. Third, our data-driven approach showed that traditionally emphasized features—such as cooling technology and gross generation—are of primary importance but can be surpassed by other, often overlooked, features when modeling WW or WC individually. The plant configuration, cooling technology, and gross generation were the most important features of WW, whereas WURM, cooling technology, and reporting month were the most important for WC. These findings can improve thermoelectric power plant management, water use reporting accuracy, and water use modeling.

## Abstract 2

This study shows the importance of power plant operations and the water use reporting methods for thermoelectric power plant management, introducing an operations-based data filtration method and investigating the importance of features for predicting reported water withdrawal and consumption.

## 1 Introduction

Thermoelectric power generation is a critical component of the U.S. energy system, contributing 73% of the country’s electricity generation.^1^ Thermoelectric power generation, however, is water-intensive, accounting for approximately 41% (503 million m3 per day) of total freshwater withdrawals in the U.S.^2^ This water intensiveness can exacerbate regional water stress, creating implications for both water management and the sustainability of the energy sector.^3−9^ Moreover, water stress poses risks to electricity generation and can lead to operational inefficiencies and potential plant shutdowns.^10,11^ The contribution of thermoelectric power plants (TPP) to water stress and the reciprocal threat water stress has on electricity generation underscores the need to understand the predictors of water use in this sector.

The cooling technology a TPP uses is the most water-intensive part of thermoelectric power generation, as it dissipates the excess heat produced during generation.^12−14^ However, different cooling technologies vary substantially in their water withdrawals (WW) and water consumption (WC). In this study, WW (million gallons) is defined as water removed from groundwater or surface water sources for use, while WC (million gallons) is the portion of water that is lost by evaporation.^2^ We use the term water use when referring to all aspects of how TPP uses water, which encompasses both WW and WC. Water withdrawal intensity (WWI) and water consumption intensity (WCI) are used to represent the volume of water associated with each unit of energy produced with units like gallons per megawatt hour (Gal/MWh). Once-through cooling systems (not including those with cooling ponds) withdraw the most water because they continuously intake fresh water and use it only once before discharge. This system possesses a lower WC as most withdrawn water returns to its source at a higher temperature.^15^ In contrast, recirculating systems using a cooling pond or cooling tower have lower WW as they reuse water within a closed cooling loop with the condenser. However, the WC is greater due to the evaporative losses within the closed system and needs to be periodically replenished.^15^ Dry cooling systems have minimal WW and WC as they rely on air instead of water using fans and heat exchanges. This cooling system is less efficient than water-based cooling technologies, which impacts plant operational costs, especially in high-temperature regions.^15^ Hybrid cooling technologies—such as once-through with cooling ponds or dry cooling systems paired with water-based induced draft cooling towers—provide flexibility to switch between dry and wet cooling as operationally needed and following local temperature conditions. These hybrid approaches vary in WW and WC based on the active cooling technology actively used.^10^ The configuration of a TPP’s cooling system has implications for WW and WC volumes, impacting both local and regional water availability and aquatic ecosystem health.^14^

Despite the importance of water to the operation of TPPs, inconsistencies in the reported plant-level water use have existed for decades.^16^ Researchers have attempted to address known data quality issues in reported water use records through statistical tests to remove outliers (e.g., refs (4, 10, 17−19)), assuming that anything outside a certain range of water use seen at similar plants is infeasible. While statistically driven, these studies did not consider whether water use estimates were reasonable or even plausible from a plant operation perspective. These approaches can potentially lead to biased WWI and WCI values by failing to capture a representative range of plant configurations and operations–i.e., filtering plausible data or including operationally infeasible records. Analysis derived from these studies’ methods may include biased data points that skew calculated water use coefficients.

Early studies on TPP water use faced data availability and accessibility challenges, impacting the capability to derive operationally robust estimates of WWI and WCI. Macknick et al.^10^ was a pivotal study that compiled previously inaccessible data sources, producing comprehensive ranges of water use estimates across multiple generator and cooling technology combinations. This effort was followed by thermodynamic assessments of TPP water use,^22^ initial assessment of public Energy Information Administration (EIA) data,^3^ and analysis of the new EIA thermoelectric cooling water data set,^4^ which was the product of the updated reporting requirements. Previous studies based on EIA data have relied on annual reported values due to the noisiness of reported subannual values. However, as data availability and quality have improved, so have the TPP water use estimates and estimation methods.^17^ Given the improved quality and availability of reported monthly data and associated metadata, researchers can now develop sophisticated analyses of the factors influencing TPP water use.

Previous studies, such as Macknick et al.^10^ and Scanlon et al.,^12^ primarily focused on cooling technology, fuel type, and generator type as the main predictors of WWI and WCI. As mentioned, early analyses were constrained by data availability, which concomitantly limited the analysis of the role that operational factors play in TPP water use. Studies by Clement et al.^20^ and Tidwell et al.^21^ explored the role of plant operations on TPP water use, suggesting the need for a more operationally grounded approach to analyze TPP water use. Further, recent studies have explored the relationships between TPP and water use in greater detail, as well as geographic and regulatory regions and different TPP technologies.^4,17^ Our study builds on previous research by reanalyzing TPP water use data from the EIA, focusing on previously under-explored factors such as a plant’s power-cooling ratio (PCR) and methods of water use reporting.^21^ We explore how factors—such as cooling technology, operational practices, geographic location, and generator technology—influence TPP WW and WC. We aim to provide an operationally grounded understanding of how these factors influence TPP WW and WC.

This study had two main objectives, each uniquely contributing to our understanding and analysis of TPP water use. First, we developed a novel filtering approach to EIA TPP water use data, employing the PCR^21^ to define plausible plant operations and obtain cleaned WW and WC values. This method differs from traditional statistical approaches^4,17^ because it offers an operationally grounded data cleaning approach and allows for the calculation of more accurate WWI and WCI values. In doing so, we aim to provide more accurate insights for researchers, data collection agencies, and policymakers, thereby contributing to developing effective sustainable water resource management strategies in the electricity sector. Second, we investigated the extent to which the PCR and methods of water use reporting, among other predictive features, drive the magnitude of TPP WW and WC. Previous studies have explored the influence of plant characteristics, such as cooling technology, location, and generator primary technology have on TPP WW and WC. However, to date, no studies have more broadly investigated the influence that plant operations and water use reporting have on TTP water use predictions.

To achieve our research objectives, we investigated the following questions: (1) how does the PCR compare to traditional statistical methods in filtering plausible ranges for TPP water use? (2) what is the spatial distribution of TPPs that are not operating efficiently? (3) how do different methods of water use reporting impact the accuracy of TPPs’ reported water withdrawal and consumption? (4) how do the combined influences of plant attributes and operations explain WW and WC in TPPs? We explain the data and methods employed to answer these questions in Section 2, while the answers to these questions are provided in Section 3. We conclude in Section 4 by summarizing our key findings and discussing the implications of our findings for power plants and the EIA, as well as those that use these data and derived water use coefficients for research, planning, and management of TPPs and water resources.

## 2 Materials & Methods

### 2.1 Data

Detailed power plant-level data, including water use, come from the EIA 860 and EIA 923 data sets. The type of plant-level data reported in the EIA 860 and 923 data sets differ.^53,54^ EIA 860 provides plant-level details about boilers, generators, cooling systems, latitude, longitude, and the configuration of boilers, generators, and the cooling system. EIA 923 details the method of how water withdrawal and consumption were measured/estimated and the temperature of the intake water in addition to the amount of water used. This water use measurement variable is called “method for flow rates” in the EIA 923 data set, hereafter called “water use reporting method” (WURM) for clarity. Beginning in 2014, the EIA merged these two data files and published the combined data online.^23^ Combined, these data sets provide a robust view of the factors that can influence water use at the plant level.

The combined EIA 860 and EIA 923 data product from the EIA served as the basis for our analysis. We considered the 6 year window from 2015 to 2020. All data and code used in this study are publicly available on HydroShare.^24^ The data set was filtered to include only TPP configurations with one cooling unit to simplify analysis and develop WW and WC values without the confounding factor of multiple cooling units. The resulting configurations consisted of TPP with (1) one cooling unit, multiple boilers, and multiple generators (1C MB MG), (2) a “simple” configuration (1C 1B 1G), (3) one cooling unit, one boiler, and multiple generators (1C 1B MG), and (4) one cooling unit, multiple boilers, and one generator (1C MB 1G). Refer to Table S1 for the count of each plant configuration type present in the processed EIA data set. This plant configuration filter allows an apples-to-apples comparison of plant configurations with single cooling systems, thereby better isolating the primary agent of plant water use. This filter enhances our study accuracy by ensuring that variations in water use intensities are attributed more directly to differences in cooling technologies and plant operations, rather than confounded by the presence of multiple cooling systems. Future studies could investigate how multiple cooling units in plant configurations influence our understanding of operational dynamics and impact WW and WC estimates.

The data were further filtered by only selecting TPPs that reported both water withdrawals and consumption, dropping duplicates, and removing records that did not report generator primary technology, cooling technology, or WURM. From the original 256,138 records, data cleaning resulted in 157,452 records of monthly reported plant data across 434 unique TPPs. Additional records were removed before analysis, as described below. Grouping records by technological, methodological, and spatial similarities further supported our analysis. This study uses the commonly reported units of million gallons to stay consistent with the EIA data sets. Plants using multiple fuel sources in the EIA data set report each type as distinct generator technology records. For example, in January 2020, the Barry, AL TPP reported 17 unique records, representing 3 conventional steam coal, 2 natural gas steam turbines, and 12 natural gas fired combined cycle generator technologies. Details explaining how reported records were categorized into the National Oceanic and Atmospheric Administration (NOAA) climate region, WURM, and cooling technology abbreviations are provided in Tables S2–S4.

### 2.2 Power-Cooling Ratio

Previous studies have approached filtering EIA data using different statistical filters to remove outliers from EIA data sets before creating water use coefficients. For example, Peer & Sanders^4^ used a four-step statistical outlier criteria to filter EIA data before calculating water use efficiencies. First, they filtered the data for only coal, natural gas, and nuclear power plants. Next, they removed plants that reported multiple fuels, prime mover, or cooling types. Then, generators with wet cooling systems with values of zero or no record of water use were removed. Finally, a statistical cutoff (*z*-score >3.5) was used to filter outliers. By contrast, De La Guardia et al.^17^ utilized a statistical filtering process that first removed TPPs with limited reported water withdrawal and consumption estimates (specifically *n* ≤ 34). Next, they assessed WWI and WCI and used a statistical cutoff (coefficient of variance >2) to remove records. Then, De La Guardia used a Pearson correlation coefficient (*r*) test to remove plants from their data set if the *r* between gross generation and water use was negative.

This study uses the PCR (γ) to filter EIA data of operationally implausible values.^21^ We use the PCR as a general proxy of plant operations that relates the operation of the plant’s generator(s) to the operation of its cooling system. This approach differs from previous studies that use statistical filters to remove outliers from the EIA data set before creating WWI and WWC coefficients. The PCR, γ, measures the relationship between the power generation system’s operational hours relative to that of the cooling system and so represents the operational efficiency of a TPP. The PCR (eq 1) was developed by Tidwell et al.,^21^ where γ is the calculated hours of power generation (hours), *E*, divided by the power plant’s hours of cooling operations (hours) *h*^c^ at plant *i*, during month *t*.
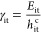
1

Because the EIA does not report the total hours of generator operations, we estimate this by calculating the quotient of gross power generation (MWh), P, and generator summer capacity (MW), C (eq 2), as was done by Tidwell et al.^21^ Here, we presume a linear proportionality between the gross power generation and operational hours. This approach provides the minimum hours required to generate the reported energy production. As such, we have confidence that removing records identified as infeasible (i.e., generator hours exceeding cooling system hours) retains the operationally plausible data in our study.
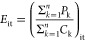
2

The log10 γ equaling 0 is interpreted as the plant’s cooling system and generators operating for equal durations. When log10 γ ≈ 1, the generator operates at 10 times the cooling duration, while log10 γ ≈ −1 indicates that the cooling system operates at 10 times greater than the generator duration. Because the cooling system must operate while the plant generates electricity, we define infeasible operating characteristics as where the number of operational hours for power generation exceeds the operational hours of cooling system (log10 γ > 0). From this logic, we set the upper bound for records in our plausible operations range at log10 γ ≈ 0. Alternatively, we define extreme operating characteristics as a cooling system operating over 10 times longer than the plant’s power generation, where the lower bound is set at log10 γ ≈ −1. This lower-bound cutoff removes the monthly records of plants potentially using more electricity than they generate, likely due to generator and boiler idling.^21^ Thus, the PCR filter creates a filtered data set of reported EIA values that fall within our proposed operationally plausible value range without the distributional tails of the extreme or infeasible TPP operations (Figure S1).

The proposed plausible range is designed to identify and exclude data points associated with infeasible (log10 γ ≥ 0) or extreme (log10 γ ≤ −1) plant operations as a means of removing operationally implausible records. Despite defining a stringent range of operational feasibility, the proposed PCR range retains 83.78% of reported monthly TPP data from the prefiltered data set (131,905 unique records). Still, there may be erroneously reported WW and WC values within our filtered data set. There is no way to identify all erroneous values without visiting each plant and taking direct and consistent measurements. However, such actions are unnecessary because our goal was to remove the records at the distribution tails representing extreme and infeasible operations to provide a cleaned data set of a plausible range of plant operations.

### 2.3 Multivariate Regression Tree

We used a multivariate regression tree (MRT) model^25^ to investigate the importance of different variables in determining the WW and WC of TPPs. MRTs are well suited to assess the multivariate influence of different plant features due to the model’s ability to handle high dimensional data with multiple predictors and its ease of interpreting factor influences. While advanced ensemble learning techniques such as bagging,^26^ boosting,^27^ random forests,^28^ or Bayesian additive regression trees^29^ may have more predictive power, this study requires the interpretability of an MRT to identify the variables driving predictions.

The MRT used reported WW and WC as target variables. WW and WC are closely related processes; by analyzing these two target variables simultaneously, we can leverage shared information between them and provide insights into their complex nonlinear relationship. The full model consisted of 12 predictor variables, which were narrowed to the 8 predictor variables that explained the vast majority of variance in water consumption and withdrawal (for the full list of model predictor variables and the simplified model, refer to Tables S5–S6). Feature reduction was done to reduce overfitting while maintaining predictive power.^30^ We justify including the log10 PCR as a predictive feature in the model as the PCR filter approach only removes the far outer tails of the data distribution and retains the central distribution of the data.

We investigated potential overfitting and identified the optimal MRT depth using root mean squared error (RMSE) and coefficient of determination (R^2^) learning curves at each MRT tree depth. Further details on evaluation of the model’s performance can be found in the Supporting Information Text S1, with details on model development and performance located in the Supporting Information Text S2 and Figure S2. We use the permutation importance index to evaluate the importance of the predictor variables in the MRT in predicting WW and WC.^31^ The permutation importance metric randomly shuffles the features used in predicting the target variables and notes the decrease in accuracy.^31,32^ We pair the permutation importance metric with the Gini importance index to compare the importance rankings when looking at WW and WC individually and combined. The Gini importance index ranks model features by how much they reduce the uncertainty of the target variables when predicting WW and WC simultaneously.^33−36^ Further detailed discussion on the two feature’ importance metrics used and their equations are provided in Text S3.

## 3 Results

### 3.1 Comparison of Thermoelectric Power Plant Data Filtering Methods

**Figure 1:**
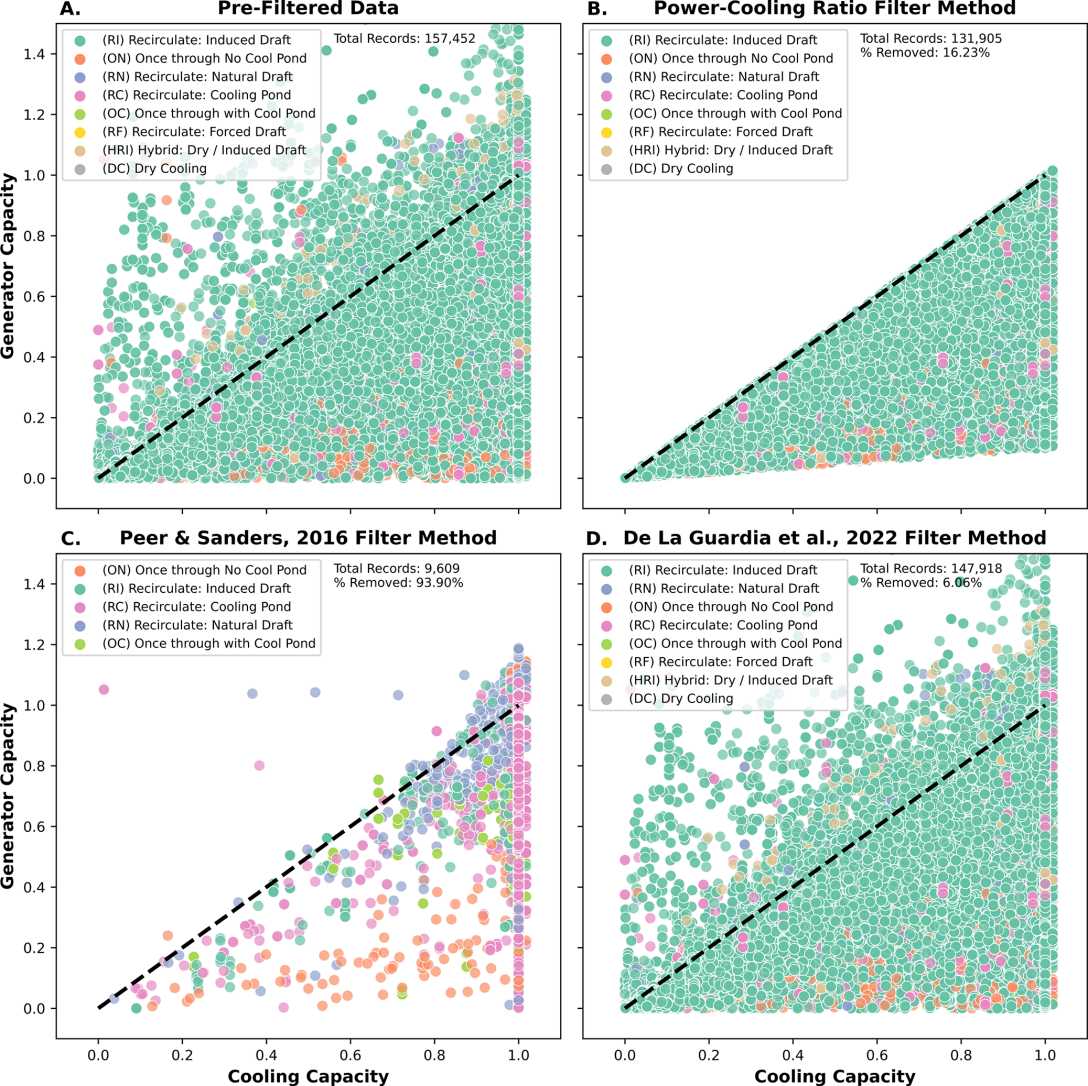
The cone of operational plausibility. Comparisons of cooling and generator capacity using different thermoelectric power plant data filtration methodologies. All plots feature a dashed line representing a 1:1 relationship between cooling and generator capacity. Values above this line would require the generator to run longer than the cooling system, which is technically infeasible. Plotted points are distinguished in color by the respective cooling technology of the plant. Values that appear to exceed the cooling capacity bound of 1.0 are values from leap years. (A) Pre-Filtered Data: relation between cooling and generating capacity with no filter methods applied. (B) Power-Cooling Ratio Filter Method (This Study): Data points were filtered out if they did not fall between 0 and −1 of the log10 power-cooling ratio criterion. (C) Peer & Sanders^4^ Filter Method: Data points were filtered using a four-step methodology that uses statistical outlier detection. (D) De La Guardia et al.^17^ Filter Method: Data points were filtered using a three-step methodology that uses summary statistics.

The method used to filter EIA TPP operational data can lead to different conclusions regarding TPPs’ water use and derived water use coefficients. As shown in Figure 1, our operationally grounded method for identifying and filtering outlier data retains between 0.89 to 13.74 times more of the reported data than statistical filtering methods used by De La Guardia et al.^17^ and Peer & Sanders,^4^ respectively. The PCR filtering method creates a cone of operationally plausible data for inclusion in the analysis (Figure 1b). Furthermore, statistical filtering methods may only retain a small sample of certain generator-cooling technology configurations, challenging generalizable water use coefficients and potentially skewing conclusions (Table S7). We find that averaged (i.e., across similar generation technology) WW and WC can be several orders of magnitude different depending on the data filtering method selected (Tables S7–S9). The average WW and WC values presented in Tables S7–S9 should not be compared to prior published values because they are a product of only four kinds of plant configurations across 434 TPPs, thus prohibiting direct comparison. These supporting tables highlight the impact of the different data filter methodologies.

Of the 434 TPPs included in the cleaned EIA data set, 46 plants (10.06%) exceeded monthly γ bounds more than 50% of the 72 month study period (refer to Table S10); 55 plants (12.67%) exceeded monthly γ bounds between 25 and 50% of the study period (18–36 months); and 75 plants (17.28%) never exceeded monthly γ bounds. Of the 48 plants that exceeded γ bounds more than 50% of the study period, 18 were nuclear power plants, representing 81% of the U.S. twenty-two nuclear power plants in the cleaned EIA data set.

We found that cooling technology influenced whether a TPP exceeded the log10 PCR (γ) bounds. Among the records outside these bounds, the top four cooling technologies were recirculating systems with induced draft cooling towers, accounting for 78.72% of out-of-bounds data and 15.64% of this technology’s records was removed by the PCR filter; recirculating systems with cooling ponds, contributing 12.9% of out-of-bounds values and 22.42% of its records filtered out; once-through systems without cooling ponds, comprising 3.75% of out-of-bounds values and 11.34% of their total records filtered; and recirculating systems with natural draft cooling towers, making up 3.33% of out-of-bounds values and had 24.13% of records using this technology removed (Table S11).

The top four generator types that exceeded the γ bounds were: natural gas-fired combined cycle generators, which accounted for 72.29% of all out-of-bounds data, with 16.06% of records for this generator type removed by the PCR filter; conventional steam coal generators, making up 9.64% of out-of-bounds values and 9.27% of its total records filtered out; natural gas steam turbines, contributing 6.8% of out-of-bounds values and had 24.39% of its records removed; and nuclear generators, which made up 5.93% of out-of-bounds data, with 77.08% of nuclear generator records removed (Table S12). These generator types represent the majority of out-of-bounds values. The natural gas fired combined cycle generators’ being the largest out-of-bounds percentage makes sense as they are the most dispatchable TPP serving as peaking plants, meaning that they are frequently turned on and off at varying generation loads to meet peak electricity demands.

**Figure 2:**
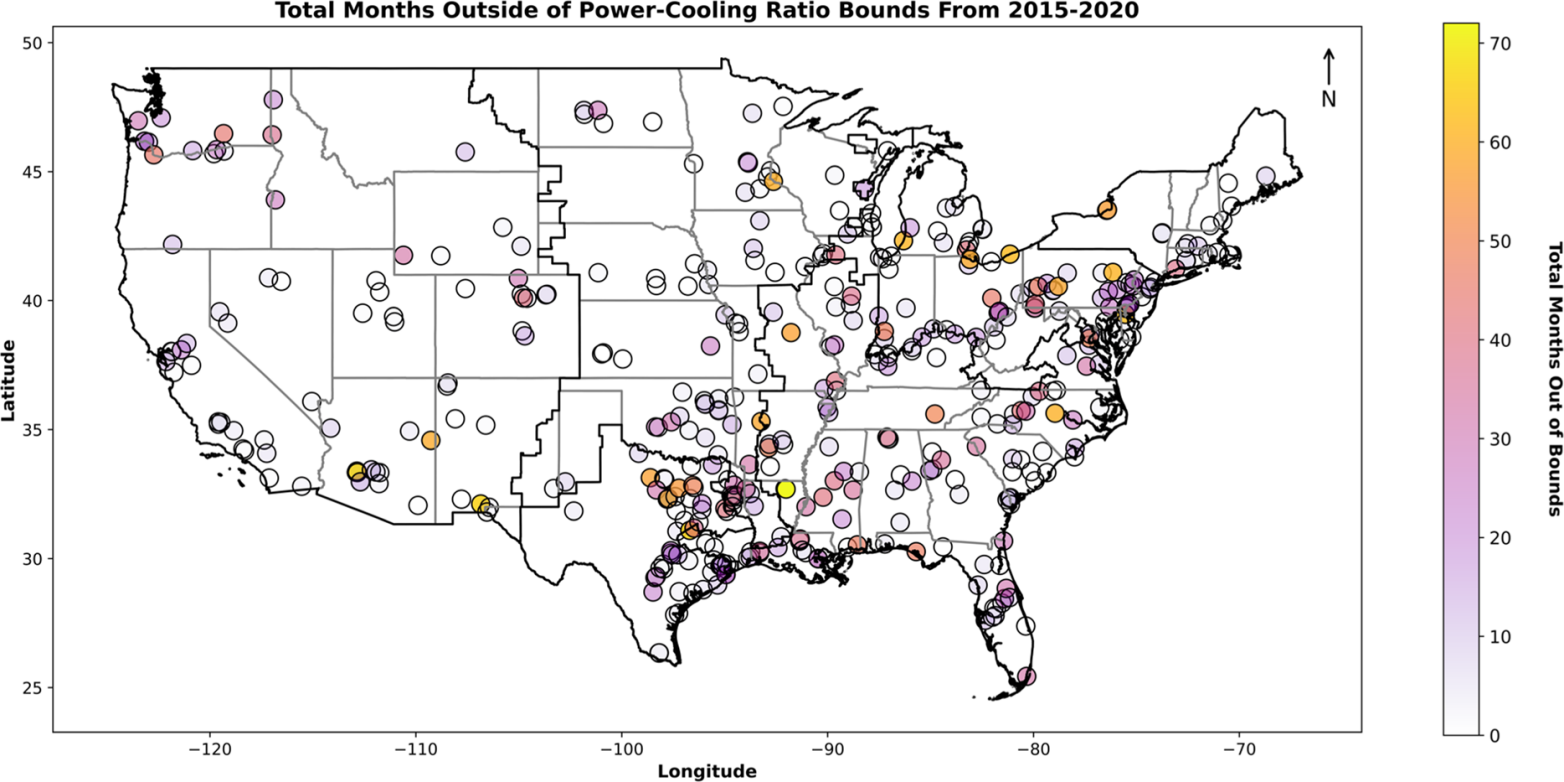
Thermoelectric power plants (points) based on the frequency they fell outside the defined log10 power-cooling ratio (γ) bounds between 2015 and 2020. The color of each point reflects the number of months with out-of-bounds power-cooling ratios. The NERC region boundaries are outlined in black, and the state boundaries are outlined in gray.

We mapped the number of months in the 72 month study period in which reported TPP data fell outside γ bounds (Figure 2). The SERC Reliability Corporation (SERC; 31%), ReliabilityFirst Corporation (RFC; 20.4%), Western Electricity Coordinating Council (WECC; 15%), and Texas Reliability Entity (TRE; 12.2%) North American Electric Reliability Corporation (NERC) regions possessed the highest number data points that exceed γ bounds. When normalized by TPP operating months (Table S13), SERC (16.8%) has the highest percentage of data exceeding PCR (γ) bounds, followed by the RFC (15.6%) and the TRE (13.6%). Many TPPs do not turn off their cooling systems because it is not efficient or cost-effective to restart the cooling system.^21^ On average, cooling systems run 13% longer than boiler systems annually, defined as the idling gap.^21^ While idling gap water use may not be a large concern for water-abundant regions such as the Southeast U.S., there was a slight increase in TPPs operating outside of γ bounds in the arid to semiarid Southwest through the study period, suggesting idling gap usage of scarce water resources.

### 3.2 Relationship between WURM and Data Quality

When the PCR is applied to the EIA data, different percentages of each WURM are removed (Table 1). The unspecified “other” WURM is the most removed WURM, with a 32.58% reduction in records after data filtering, followed by permitted values (26.59%), estimated methods (17.36%), measured methods (15.07%), and not reported methods (12.09%). While Table 1 reports how the PCR filter affects broad WURM categories, Table S14 provides a breakdown of the total count and percentage removal of individual WURMs reporting pre- and post-PCR filtration.

**Table 1:** Comparing Data Removal by the Power-Cooling Ratio Filter by Aggregated WURM Categories^a^

water use reporting methods	total data records		
	raw data	power-cooling ratio	% Removed
estimated methods	69,496	57,432	17.36%
measured methods	69,343	58,893	15.07%
other (provided explanation in schedule 9)	3026	2040	32.58%
permitted value, not measured	1113	817	26.59%
no reported method	14,474	12,723	12.09%

a The total number of original records are reported alongside the number of records after applying our data filtering procedure using the power-cooling ratio. The percentage of removed records is reported in the last column for each WURM.

From Table 1, we observe that measured methods account for a similar number of records as estimated methods yet retain a higher percentage of data postfiltering. This result indicates that efficient plant operation and plausible reported water use records are more affiliated with TPPs using measured methods instead of estimated, other, permitted, or unreported methods. We observe that estimated reporting methods make up the majority of WW volumetric values pre- and post-PCR filter (Table S15; 64.12% and 58.68%, respectively). The decrease in estimated WW total volumes pre- and post-PCR filter is because many removed records were extremely large outliers. In contrast, measured reporting methods comprise most of the total WC volumetric values pre- and postfiltering (Table S15; 67.26% and 68.64%, respectively). This finding indicates that the WURM may be more influential in capturing accurately reported WC values than WW values. Furthermore, even when accounting for differences in electricity generation, the mean monthly plant level WW and WC values can differ by orders of magnitude depending on the WURM.

Further analysis revealed statistically significant differences between WW and WC volumes across the various aggregated WURMs before and after applying the PCR filter, as indicated by the analysis of variance (ANOVA) and Games-Howell posthoc test results (refs (35)–^37^; for a detailed description of the ANOVA and Games-Howell posthoc test alongside their results, see Text S4 and Tables S16–S18). The ANOVA results indicated a statistically significant effect of WURMs on WW and WC volumes (*p* < 0.01), suggesting that WURMs contribute to the observed variations in reported water use data. Games-Howell posthoc tests further highlighted these effects, where estimated water withdrawal values consistently exceeded measured and permitted values, indicating that estimation methods may overstate water needs compared to direct measurements. In contrast, WURMs such as “no reported method” or “other” represented much lower volumes, implying potential under-reporting, data omissions in these methods, or a propensity to be associated with smaller TPPs. These findings highlight the importance of consistent and accurate reporting practices in reporting WW and WC values, as discrepancies among methods can introduce substantial variability in reported volumes (see Tables S16–S18).

### 3.3 Importance of Features in Predicting WW and WC

**Figure 3:**
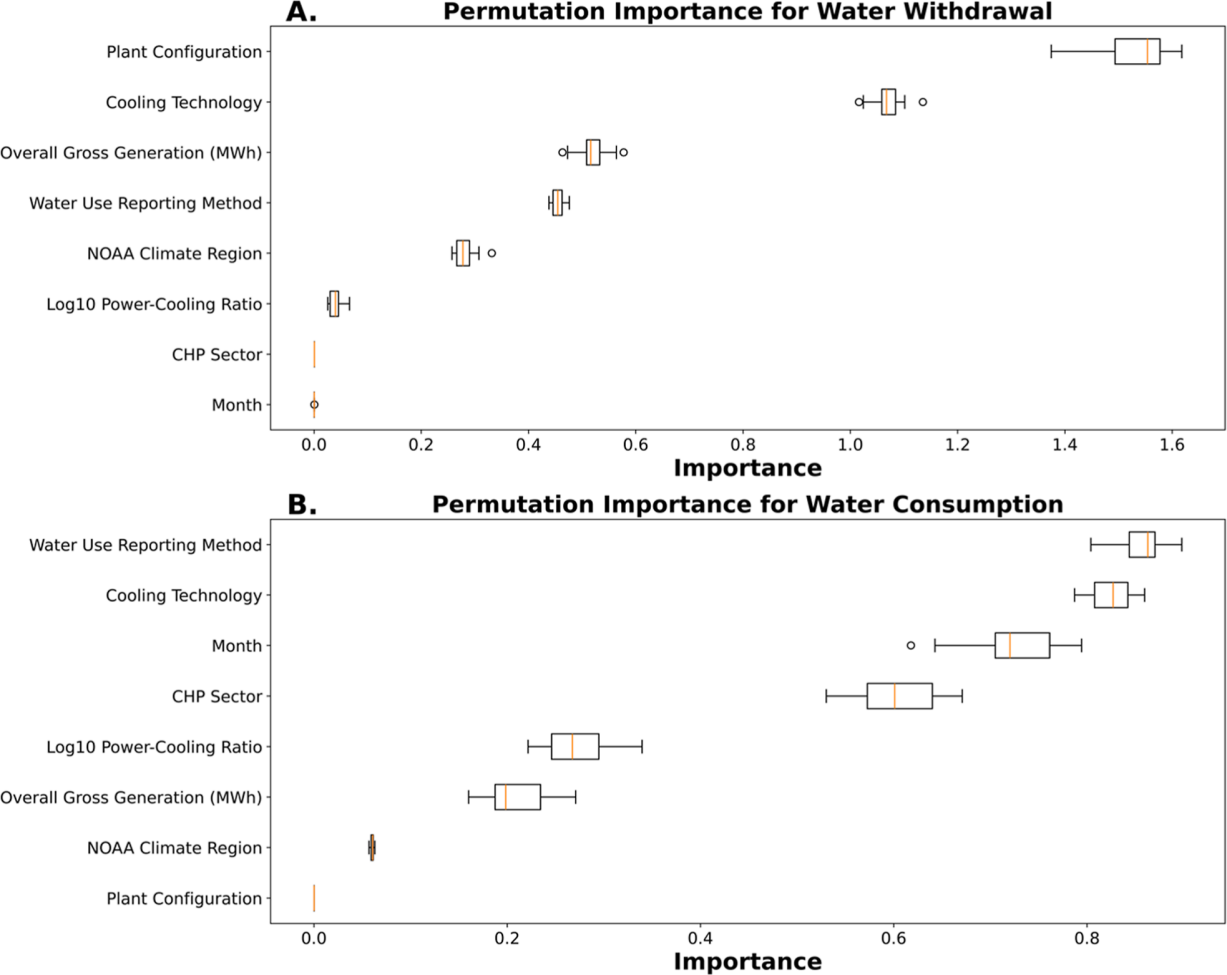
Permutation importance metrics of input features for predicting (A) water withdrawal and (B) water consumption based on the pruned Multivariate Regression Tree model and the simplified list of model features. Each boxplot represents the distribution of permutation importance values across the 10 cross-validation folds for each feature. The vertical line inside the box represents the median, while the box edges represent the 25th and 75th percentiles. Whiskers extend up to 1.5 times the interquartile range, and points outside the whiskers are plotted as outliers. Higher median importance values indicate a stronger influence of that feature on the model predictions.

The permutation importance was used to rank the predictive power of features by measuring the decrease in the MRT performance when the features were randomly shuffled. Permutation importance (Figure 3) was run individually to predict reported WW and WC. The permutation importance analysis revealed distinct patterns in feature importance when predicting TPP reported WW and WC. When the simplified list of TPP attributes was assessed together, we found that certain features were important to both WW and WC, and other features that were more important when predicting reported WW or WC individually (Figure S3).

Cooling technology and WURM emerged as influential features for WW and WC. Cooling technology held high significance for WW (mean importance of 1.07) and WC (mean importance of 0.82), indicating that different cooling configurations primarily drive TPP WW and WC, which is supported by the literature.^10−15^ Additionally, WURM was similarly important for both target variables, with mean importance values of 0.45 for WW and 0.85 for WC. This highlights the impact of how TPPs report their water play in predicting accurate WW and WC values. For WW, plant configuration is the most important feature (mean importance of 1.54), indicating that the operational configuration of a TPP (i.e., if it has multiple cooling systems, boilers, or generators) is very influential in the volume of WW from their water sources. Furthermore, overall gross generation (MWh) and the NOAA climate region were more important to WW (mean importance of 0.51 and 0.28, respectively) than to WC. This could be due to the amount of electricity generated, and the regional climate conditions dictate the TPP’s cooling requirements and thus influence the withdrawal volumes required.

By contrast, month and the combined heat and power (CHP) plant sectors, which had respective mean importance of 0.73 and 0.6, were among the most influential plant features alongside WURMs (mean importance of 0.85) and cooling technology (mean importance of 0.82), which were the most important features. This temporal and sectoral impact on WC likely reflects the seasonal variations in water evaporation rates alongside the sector-specific practices and water demands. The log10 PCR contributed more to WC (mean importance of 0.27) than WW (mean importance of 0.03), suggesting that a plant’s cooling-to-generation efficiency impacts water consumption through evaporation. This finding is also supported by the measured methods representing a larger volume of WC total values than all alternative reporting methods (Table S15). These findings underscore the important nuanced features in predicting reported WW and WC that have not been consistently considered in previous studies.

When looking at the relative Gini importance (*G*) ranking of features for predicting reported WW and WC together, we observe that the cooling technology (*G* = 0.36), overall gross generation (MWh) (*G* = 0.26), WURM (G = 0.14), and plant configuration (*G* = 0.1) are the top 4 features (Figure S3), values are ranked with the sum of their values equaling 1. This alternative importance ranking, though performed on WW and WC together, reinforces the main findings of feature importance from the permutation importance metric. The permutation importance of cooling technology, overall gross generation (MWh), WURM, plant configuration, month, CHP sector and log10 PCR to a TPP’s WW and WC remains robust across different filtering bounds. Each of these features consistently influence the MRT outcomes and remain among the top four in importance when looking at different PCR filter value bounds. However, their importance ranking order changes when the PCR filtering bounds are adjusted from log10 γ = 0 to −1 (our basis) to 0 to −4 (indicating that the cooling systems operate up to 10,000 times more than the generator systems).

## 4 Discussion

We reanalyzed self-reported monthly U.S. TPP WW and WC data to study the underlying predictors of TPP water use. To achieve this, we deployed a unique data filtering approach based on TPPs operational characteristics. While the PCR itself is not a new equation or model feature, the application of the PCR as a data filter for EIA data sets is novel.^21^ The PCR’s performance in removing implausible or extreme data, compared to previous statistical filter approaches (e.g., refs (4, 17)), sets it apart as the current best-performing TPP data filtering methodology, a key contribution of this study (Figure 1).

The PCR filter produces an operationally plausible plant-level water use data set that revealed potential systematic biases. For instance, TPPs with recirculating cooling systems disproportionately reported infeasible or extreme operational conditions, suggesting a technological linkage associated with misreported plant operations. These reporting inconsistencies may be due to unclear water use reporting guidelines, which would point to the need for clearer reporting instructions.^18,19^ Additionally, we find that SERC, RFC, WECC, and TRE TPPs report extreme or infeasible plant operations more often than other NERC regions. Notably, SERC and TRE have the greatest share of estimated water withdrawal and consumption records (as opposed to metered or permitted) compared to all other NERC regions. Furthermore, TPPs in the arid to semiarid Western U.S. report operating cooling systems longer (10x or more) than generator systems, a notable finding for a region amid a multidecadal megadrought since such operations increase WW and WC without a corresponding increase in electricity production.^55^

A second novel and key contribution of this study is finding statistically significant differences in TPP WW and WC volumes between different WURMs. We found TPPs that use measured WURMs instead of estimated or alternative WURMs are less likely to report extreme or infeasible operations. However, if we were to censor the post-PCR filtered EIA data set to only contain “measured” WURMs, the data set would decrease to 58,893 records (44.65% of the operationally plausible 131,905 records), drastically reducing the available data for analysis and limiting the impact of potential findings. WURMs were identified as statistically significant in their differences for WW and WC volumes in pre- and post-PCR filtering (Table S16), highlighting that estimated methods may overstate or understate TPP WW and WC compared to measured methods (Tables S17 and S18). For WW, measured WURMs have less influence on capturing operationally plausible values. Estimated WURMs represent 58.68% of the total WW volumetric value after applying the PCR filter (down from 64.12% prefilter; Table S15), highlighting how operational decisions regarding WURM can influence variations in reported TPP water use values.

Despite being an effective filter for obtaining operationally plausible data, the PCR has limitations. First, we utilized a static factor, reported nameplate summer capacity, as the basis for calculating plant generator system monthly operational hours. The nameplate summer capacity is defined as the maximum amount of electricity a generator can produce during the summer months without exceeding its design thermal limits. Prior studies have found that monthly generator capacity varies over time and can be greater than the reported nameplate summer capacity,^38−40^ but, unfortunately, the EIA thermoelectric cooling water data set does not report monthly values of a plant’s capacity.^23^ The EIA-860M data set showed promise by reporting the nameplate capacity, summer generator capacity, and winter generator capacity.^40^ However, these values almost always remained static across each reported month. Thus, the PCR may label a plant that exceeds its nameplate summer capacity as infeasible despite being a plausible operation due to variance in monthly generator capacity. Exceedance of nameplate summer capacity likely explains many of the out-of-bounds operations of nuclear power plants. Of 1514 out-of-bounds monthly records across all nuclear power plants, 1435 records (94.8%) had log10 PCR scores between 0.0 and 0.05. One interpretation of this finding is that the generator operated slightly longer than the cooling system; however, an alternative, more plausible, explanation is that the actual capacity of the generator is greater than the provided nameplate summer capacity and that generation and cooling hours were identical.

It should be noted that TPP electricity generation is subject to stricter reporting requirements than other plant operations, including water use, and while our filtering methods capture many of the likely misreported values of other plant operations, it is likely that some erroneous data remains. Given the inherent uncertainty in some data records, we evaluated different PCR bounds and found that the key predictors of WW and WC remained robust against different MRT model configurations. The operationally plausible bounds applied in this study are broad, because they cover multiple generator and cooling system combinations. This framework is flexible in that it can be tailored to specific operational combinations to better capture operationally plausible data better. Future studies that investigate additional generator and cooling system combinations could help further define the cone of operationally plausible data (Figure 1) for predicting TPP reported WW and WC.

Another key contribution of this research beyond introducing the data filtering method and the WURM used is the new insights into the predictors of WW and WC for TPP. As expected, cooling technology and overall gross generation (MWh) are the two most influential features for predicting reported WW and when predicting for both reported WW and WC together. A novel finding was the influence of WURMs on WW, and even more so on WC, highlighting the significance of accurately measuring reported flows for operationally plausible values. This finding reinforces the previously noted statistically significant differences between WURMs on TPP WW and WC reported volumes (Tables S16–S18). TPP configuration, identified as the most influential factor in predicting reported WW, has not previously been recognized, and this may also be why PCR is undervalued. This high importance tied to TPP configuration could be due to specific configurations and operations of each TPP and the nonlinear cycling gap where not all boilers or generators are operating, but the cooling system continues to operate.^21^ Notably, this study only examines plant configuration with single cooling systems. This limitation suggests that plant configurations with multiple cooling systems could have different implications for WW and WC predictions, potentially adding further complexity to the influence of PCR. Additionally, the log10 PCR proved to moderately influence WC, indicating that the cooling-to-generation operational efficiency plays a role in consumptive water use at a TPP. The importance of month and NOAA climate region for predicting reported WC highlights the seasonal and regional variability in electricity demand and ambient temperature on a TPP.^4,38,39^ The identified feature’s importance specifically relates to this study’s MRT model; different model configurations may produce different rankings of feature importance. However, we do not expect different model configuration feature important rankings to be fundamentally different from what this study found. The MRT model’s identified feature importances for WW and WC builds on prior studies that highlighted TTP water use determinants and will inform future models of TPP WW and WC to be more accurate in their predictions.^10,12,17−21^

Operational and measurement aspects are often not included in broad reviews of TPP water use. However, narrower studies revealed that operational decisions and management strongly influence nuclear TPP water use.^41−45^ These plants operate under strict regulatory constraints, likely explaining why 1885 (96%) of the 1964 nuclear records lie within a narrow PCR range of 0.05 to −1, reflected in the highly consistent EIA-reported plant operational efficiencies (Figure S4). In contrast, other TPPs, subject to fewer regulations, can respond more flexibly to load demand changes, likely resulting in the greater variability in reported water use when compared to nuclear TPPs. Given that human error has contributed to production losses, maintenance failures, misreported data, major disasters, and both latent and active failures, understanding how TPP operational decisions affect TPP water use is critical for more robust predictive models and policies.^46−52^

Noise and outliers in the unfiltered EIA data limited the MRT model’s insights, underscoring the need for a robust filtering method—such as the PCR filter applied in this study—to remove potentially erroneous data. We recommend that future researchers adopt the PCR filter to ensure TPP data reflect operationally plausible values, thereby providing more reliable analyses. Moving forward, cooling technology and gross generation could remain as primary features in TPP water-use models, as they most strongly influence both reported WW and WC. However, WURM, log10 PCR, month, and CHP sector could be included when predicting TPP WC, while plant configuration, WURM, and NOAA climate region could be considered when predicting TPP WW.

We also hypothesize that intake average water temperature may be important when predicting reported WW and WC values since higher average water intake temperatures reduce cooling efficiency and require more water to achieve adequate cooling. This study did not include this feature due to data limitations—58,355 (44.24%) post-PCR filter records were missing intake average water temperatures, and imputing these values could skew MRT results. Future models could include this feature if data availability improves.

This study underscores the challenges associated with data collection and reporting methods have in the analysis of TPP water use. Our findings emphasize the importance for both the EIA and TPP operators to develop and implement standardized guidelines for measuring water use at TPPs. Enhanced accuracy and consistency in water use reporting could significantly improve our understanding of TPP water use. High-quality data, combined with effective data filtering techniques, can help ensure that reported figures accurately reflect how TPPs utilize water, thereby strengthening our ability to project future water needs for electricity generation.

## Data Availability

The data sets and code used in this article are publicly available through CUAHSI Hydroshare at http://www.hydroshare.org/resource/aa10f4621be84c01acfc82db57b5075b. The data sets used in this study were derived from the U.S. Energy Information Administration and are in the public domain (https://www.eia.gov/electricity/data/water/; https://www.eia.gov/electricity/data/eia923/; https://www.eia.gov/electricity/data/eia860/).

## Supporting Information Available

Model performance metrics; model learning curves; model feature importance metrics; ANOVA and Games-Howell tests on water use reporting methods. Additionally, the Supporting Information includes figures showing: the distribution of log10 power-cooling ratio values for each type of water use reporting method; the model learning curves with error metrics across tree depth; the Gini importance index of model input features; the log10 power-cooling ratio vales for each type of generator primary technologies. It also includes: Tables S1 to S6 defining the explanatory features included in the model; Tables S7 to S9 comparing the different filter methods’ impact on unique generator-cooling combination data; Tables S10 to S18 showing the impact of the water use reporting method and power-cooling ratio on the EIA data set (PDF)
