# Supplementary material for: Impact of Thermoelectric Power Plant Operations and Water Use Reporting Methods on Thermoelectric Power Plant Water Use
Source: Environ Sci Technol. 2025 Feb 27;59(9):4482–92. doi: 10.1021/acs.est.4c02024 (PMC11912314; doi:10.1021/acs.est.4c02024)
Supplement: Supplementary file 1 — es4c02024_si_001.pdf [file es4c02024_si_001.pdf]

Supporting Information

The Impact of Thermoelectric Power Plant Operations and Water Use Reporting Methods on Thermoelectric Power Plant Water Use

Eric Sjöstedt [1], Richard Rushforth [1], Vincent Tidwell [2], Melissa Harris [3], Ryan McManamay [4], Landon Marston [*,5]

[*] Corresponding Author: lmarston@vt.edu
[1] School of Informatics, Computing, And Cyber Systems, Northern Arizona University, Flagstaff, AZ, 86011, USA
[2] Pacific Northwest National Laboratories, Richland, WA, 99354, USA
[3] U.S. Geological Survey, Lower Mississippi-Gulf Water Science Center, Nashville, TN, 37211, USA
[4] Department of Environmental Science, Baylor University, Waco, TX, 76706, USA
[5] Department of Civil & Environmental Engineering, Virginia Tech, Blacksburg, VA, 24061, USA

Summary of Contents:
Number of Pages: 29
Number of Texts: 4
Number of Figures: 4
Number of Tables: 18

Table of Contents

Text S1 - Multivariate Regression Tree Performance Metrics.................................. S6

Text S2 - Multivariate Regression Tree Learning Curves S7

Text S3 - Multivariate Regression Tree Feature Importance Metrics S9

Text S4 - ANOVA and Games-Howell Tests of Water Use Reporting Methods....... S25

Figure S1 - Boxplots of the log10 power-cooling ratio values plotted for each water use reporting method. The dotted red lines represent the 0 to -1 log10 power-cooling ratio value bounds used to filter the EIA dataset. The box spans from the 25th percentile (Q1) to the 75th percentile (Q3), with the median (50th percentile) shown as the vertical line inside the box. The Whiskers extend up to 1.5 times the interquartile range beyond Q1 and Q3 and log10 power-cooling ratio values lying outside this range are plotted as outliers. S5

Figure S2 - Model learning curves using error metrics across varying tree depths. The pruned tree depth of 7 for the model is shown with the vertical dashed line. A) Model performance evaluated using Root Mean Squared Error (RMSE) for the test (blue), training (orange), and cross-validation (purple) scores. B) Model performance evaluated using the Coefficient of Determination (R^2) for the test (blue), training (orange), and cross-validation (purple) scores................................. S7

Figure S3 - Gini importance index of input features for the pruned Multivariate Regression Tree Model (4). Here, the target variables, water withdrawals (WW) and water consumption (WC), are evaluated together... S10

Figure S4 - Comparison of log10 power-cooling ratio and the generator technology data boxplots across the entire prefiltered dataset. The box spans from the 25th percentile (Q1) to the 75th percentile (Q3), with the median (50th percentile) shown as the vertical line inside the box. The Whiskers extend up to 1.5 times the interquartile range (IQR) beyond Q1 and Q3 and log10 power-cooling ratio values lying outside this range are plotted as outliers. S11

Table S1 - The types of thermoelectric power plant configurations used in this study and the count of each type in the processed EIA dataset. The plant configurations consisted of thermoelectric power plants with 1) one cooling unit, multiple boilers, and multiple generators (1C MB MG), 2) a “Simple” configuration

(1C 1B 1G), 3) ONE COOLING UNIT, ONE BOILER, AND MULTIPLE GENERATORS (1C 1B MG), AND 4) ONE COOLING UNIT, MULTIPLE BOILERS, AND ONE GENERATOR (1C MB 1G)....................... S12

TABLE S2 - NOAA CLIMATE REGIONS BROKEN DOWN BY THE STATES INCLUDED WITHIN EACH REGION (6). S13

TABLE S3 - ENERGY INFORMATION ADMINISTRATION FORM-923 WATER USE REPORTING METHODS AGGREGATED INTO GROUPS OF ESTIMATED, MEASURED, PERMITTED, OTHER, AND NOT REPORTED (7). COUNT REFERS TO NUMBERS OF UNIQUE PLANTS REPORTING A GIVEN METHOD OF WATER USE REPORTING. A SINGLE PLANT CAN REPORT MULTIPLE DIFFERENT METHODS OF WATER USE REPORTING THROUGH THE STUDY PERIOD................ S14

TABLE S4 - ENERGY INFORMATION ADMINISTRATION REPORTED COOLING TECHNOLOGY CATEGORIES AND AN ADAPTATION OF EIA ABBREVIATIONS USED (8)................ S15

TABLE S5 - MULTIVARIATE REGRESSION TREE FULL MODEL OF PREDICTOR FEATURES AND THEIR DEFINITIONS. S16

TABLE S6 - MULTIVARIATE REGRESSION TREE SIMPLIFIED MODEL OF PREDICTOR FEATURES AND THEIR DEFINITIONS. S16

TABLE S7 - A TABLE OF ALL GENERATOR AND COOLING TECHNOLOGY COMBINATIONS FROM RAW DATA SHOWING HOW THE TOTAL COUNTS OF UNIQUE GENERATOR AND COOLING TECHNOLOGY CONFIGURATIONS CHANGED WHEN IN THE PREFILTERED FORM, AFTER THE PEER & SANDERS (9) FILTER, AFTER THE DE LA GUARDIA ET AL. (10) FILTER, AND AFTER THIS STUDY'S POWER-COOLING RATIO FILTER. S17

TABLE S8 - A TABLE OF ALL GENERATOR AND COOLING TECHNOLOGY COMBINATIONS FROM RAW DATA SHOWING HOW THE MEAN WATER WITHDRAWAL (MILLION GALLONS) OF UNIQUE GENERATOR AND COOLING TECHNOLOGY CONFIGURATIONS CHANGED WHEN IN THE PREFILTERED FORM, AFTER THE PEER & SANDERS (9) FILTER, AFTER THE DE LA GUARDIA ET AL. (10) FILTER, AND AFTER THIS STUDY'S POWER-COOLING RATIO. S18

TABLE S9 - A TABLE OF ALL GENERATOR AND COOLING TECHNOLOGY COMBINATIONS FROM RAW DATA SHOWING HOW THE MEAN WATER CONSUMPTION (MILLION GALLONS) OF UNIQUE GENERATOR AND COOLING TECHNOLOGY CONFIGURATIONS CHANGED WHEN IN THE PREFILTERED FORM, AFTER THE PEER & SANDERS (9) FILTER, AFTER THE DE LA GUARDIA ET AL. (10) FILTER, AND AFTER THIS STUDY'S POWER-COOLING RATIO. S19

Table S10 - A list of all power plants with reported out-of-bounds operations for 50% or more of the study period (36 months or more) with their Plant ID, name, state and summed mean number of out-of-bounds operations reported. S20

Table S11 - The counts of each cooling technology pre- and post-PCR filter application to the EIA dataset alongside the percentage of data removed by the PCR filter. S21

Table S12 - The counts of each generator primary technology pre- and post-PCR filter application to the EIA dataset alongside the percentage of data removed by the PCR filter. S21

Table S13 - A table of normalized log10 power-cooling ratio out of bounds percentages for each North American Electric Reliability Corporation (NERC) Region (11). S22

Table S14 - Comparison of total data records and percentage removed of unique and aggregated water use reporting methods for the raw data vs. power-cooling ratio filtered data. S23

Table S15 - Comparison of the percentage of total water withdrawal (left) and percentage of total water consumption (right) volume reported by different water use reporting methods for both the raw data and the filtered data. Note that each column equals 100%. S24

Table S16 - Comparison of ANOVA test results between aggregated water use reporting methods and water withdrawal and consumption both pre-and post-power-cooling ratio filter application. S25

Table S17 - Comparison of Games-Howell post-hoc test results between aggregated water use reporting methods and water withdrawal (million gallons) for pre-and post-power-cooling ratio filter application. S27

Table S18 - Comparison of Games-Howell post-hoc test results between aggregated water use reporting methods and water consumption (million gallons) for pre-and post-power-cooling ratio filter application. S27

Supporting Information References S28

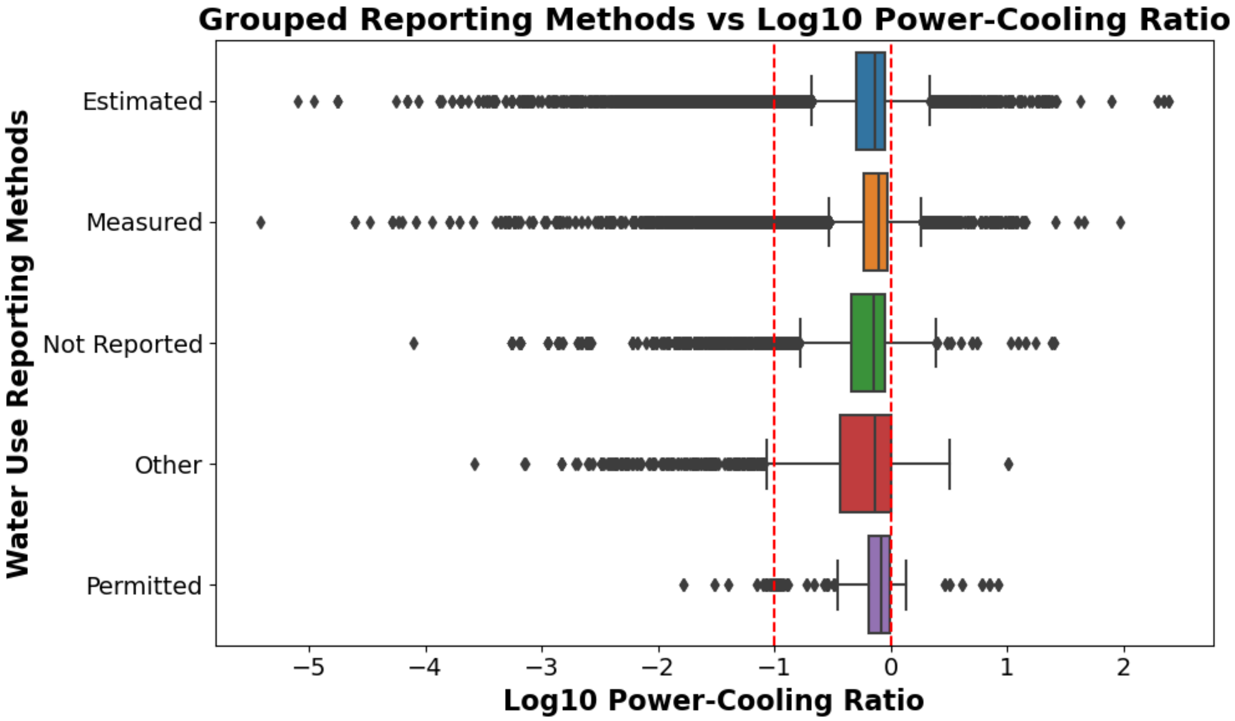

Figure S1 **-** *Boxplots of the log10 power-cooling ratio values plotted for each water use reporting method. The dotted red lines represent the 0 to -1 log10 power-cooling ratio value bounds used to filter the EIA dataset. The box spans from the 25th percentile (Q1) to the 75th percentile (Q3), with the median (50th percentile) shown as the vertical line inside the box. The Whiskers extend up to 1.5 times the interquartile range beyond Q1 and Q3 and log10 power-cooling ratio values lying outside this range are plotted as outliers.*

Text S1 - Multivariate Regression Tree Performance Metrics

The performance of the multivariate regression tree (MRT, 1) was evaluated using two metrics, root mean squared error (RMSE; Equation S1) and the coefficient of determination (R^2; Equation S2). RMSE quantifies the difference between the observed *i* and the predicted values by subtracting the actual observed value y_i from the predicted value $\hat{y}_i$, and squaring the difference. Then the square root is taken of the sum of the squared difference across all observations *n*.

$$RMSE = \sqrt{\frac{1}{n}\sum_{i=1}^{n}(\hat{y}_i - y_i)^2} \quad \text{Equation S1}$$

Lower the RMSE values indicate the better model fits; however, RMSE values are influenced by the scale of the target variable used. In this study, water withdrawal and water consumption are target variables with a wide range of low and high values (i.e., 1-140,328 and 1-27,777 million gallons respectively), so the resulting RMSE will be large. This study paired RMSE with the R^2 metric which indicates how well the variation of predictor variables can predict target variables. The metric is calculated as the sum of squared differences between the observed values y_i and the predicted values $\hat{y}_i$ over the sum of squared differences between the observed value y and the mean value for the dependent variable μ.

$$R^2 = 1 - \frac{\Sigma_i(y_i - \hat{y}_i)^2}{\Sigma_i(y_i - \mu)^2} \quad \text{Equation S2}$$

R^2 is dimensionless, not influenced by the scale of data and is a relative measure due to including a proportion of variability explained. It is interpreted between the values of 0 and 1, with 1 implying a perfect fit and the model explaining 100% of the variance present in the data. An R^2 value of 0 would imply that the model explains 0% of the variance in the dataset.

Text S2 - Multivariate Regression Tree Learning Curves

We used an MRT to determine the importance of different features in predicting power plant water withdrawals and consumption. The model underwent an 80% training and 20% testing split of the dataset with a 10-fold cross-validation approach. We determined the optimal tree depth to prune the MRT for prediction accuracy by using root-mean-square error (RMSE) and coefficient of determination (R^2) learning curves for each tree depth, allowing us to diagnose appropriate model performance across MRT evolutions before overfitting occurs. Next, we compared well-performing models on how well they fit training data using the Akaike Information Criterion (AIC; 2), which helped us identify the most predictive model with the fewest independent variables. We found that the optimal tree depth of the MRT was 7 layers. Pruning the MRT to this depth was selected following evaluation and comparison of RMSE and R^2 metrics across training, testing, and 10-fold cross-validation splits (Figure S2A & S2B).

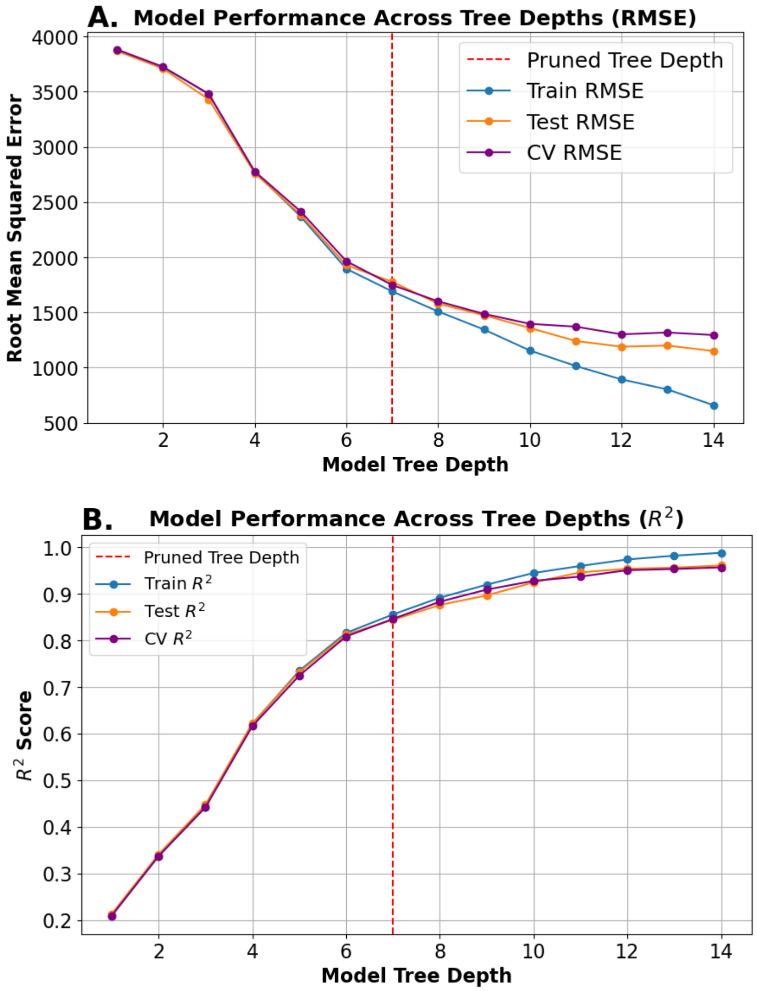

Figure S2 *- Model learning curves using error metrics across varying tree depths. The pruned tree depth of 7 for the model is shown with the vertical dashed line. A) Model performance evaluated using Root Mean Squared Error (RMSE) for the test (blue), training (orange), and*

cross-validation (purple) scores. B) Model performance evaluated using the Coefficient of Determination (R^2) for the test (blue), training (orange), and cross-validation (purple) scores.

The MRT was pruned to a depth of 7 to reduce the complexity of the model tree structure and avoid overfitting of the model to the data. The MRT performance at a depth of 7 resulted in RMSE scores of 1,677 (Training), 1,752 (Testing), and 1,750 (Cross Validation) in units of million gallons. In Figure S1A, we can observe that after the tree depth of 7 the testing and training RMSE begin to diverge, suggesting overfitting beyond this depth. R^2 scores of 0.859 (Training), 0.849 (Testing), and 0.848 (Cross Validation) support this observation, where after the tree depth of 7 the scores are asymptotic towards a score of 1 (Figure S1B). In summary, the MRT's RMSE and R^2 scores give insight into model robustness in its predictive accuracy, and 7 being the optimal tree depth to minimize overfitting while maintaining predictive performance. This pruned model achieved an AIC score of 539.87.

To reduce the complexity of the MRT, we trimmed this model to the identified 8 most influential predictive features as identified by the permutation importance metric and the Gini importance index (3, 4). The included features were: CHP sector, cooling technology, log10 power-cooling ratio, month, NOAA climate region, overall gross generation, plant configuration, and water user reporting method. A MRT depth of 7 with a reduced number of features resulted in RMSE scores of 1,690 (Training), 1,777 (Testing), and 1,748 (Cross Validation) in million gallons units, and R^2 scores of 0.856 (Training), 0.844 (Testing), and 0.846 (Cross Validation). This reduction of predictive features used by the MRT resulted in a similar AIC score of 527.93. By simplifying the model to only include 8 influential features for predicting water withdrawal and consumption, we have reduced the AIC score which indicated an improved model, the tradeoff was slight reductions in R^2 and RMSE predictive accuracy. These changes suggest that the simplified MRT balances predictive power with interpretability and generalizability that avoids overfitting, while still capturing the core relationships among the data.

Text S3 - Multivariate Regression Tree Feature Importance Metrics

We used the Gini importance index (*G*; Equation S3; 4) to understand the impact of the predictor variables on the model's predicted outputs for water withdrawal and water consumption together. The Gini importance index can rank features by their predictive power and identify the most important features for a given model. A higher Gini importance index means that the feature has a greater impact on reducing the uncertainty about the target variable. The Gini importance index, *G*, is calculated as a measure of total variance across classes *K*. In this context, each predictor variable used in the MRT model is represented as a class, referred to as *K*. With $\hat{p}_{mk}$ representing the proportion of observed training values in the mth region from class *K*.

$$G = \Sigma_{K=1}^{K} \hat{p}_{mk}(1 - \hat{p}_{mk}) \quad \text{Equation S3}$$

While effective at identifying feature importance in tree-based algorithms, the Gini importance index has been found to be biased towards features with more categories or levels (5). To mitigate potential biases in feature importance identification, we calculated the permutation importance for each feature and compared it to the Gini importance index. The permutation importance (PI; Equation S4; 3) is calculated by randomly shuffling the values of a feature *i* and measuring the decrease in the model prediction accuracy from the original model.

$$PI_i = Accuracy_{Original} - Accuracy_{Permuted\ i} \quad \text{Equation S4}$$

This approach helps identify crucial features that might not affect the MRT structure but are vital for its predictive accuracy. Used together, these metrics provide a more balanced and comprehensive insight to the MRT model's feature importance, enhancing the model's interpretability and reliability.

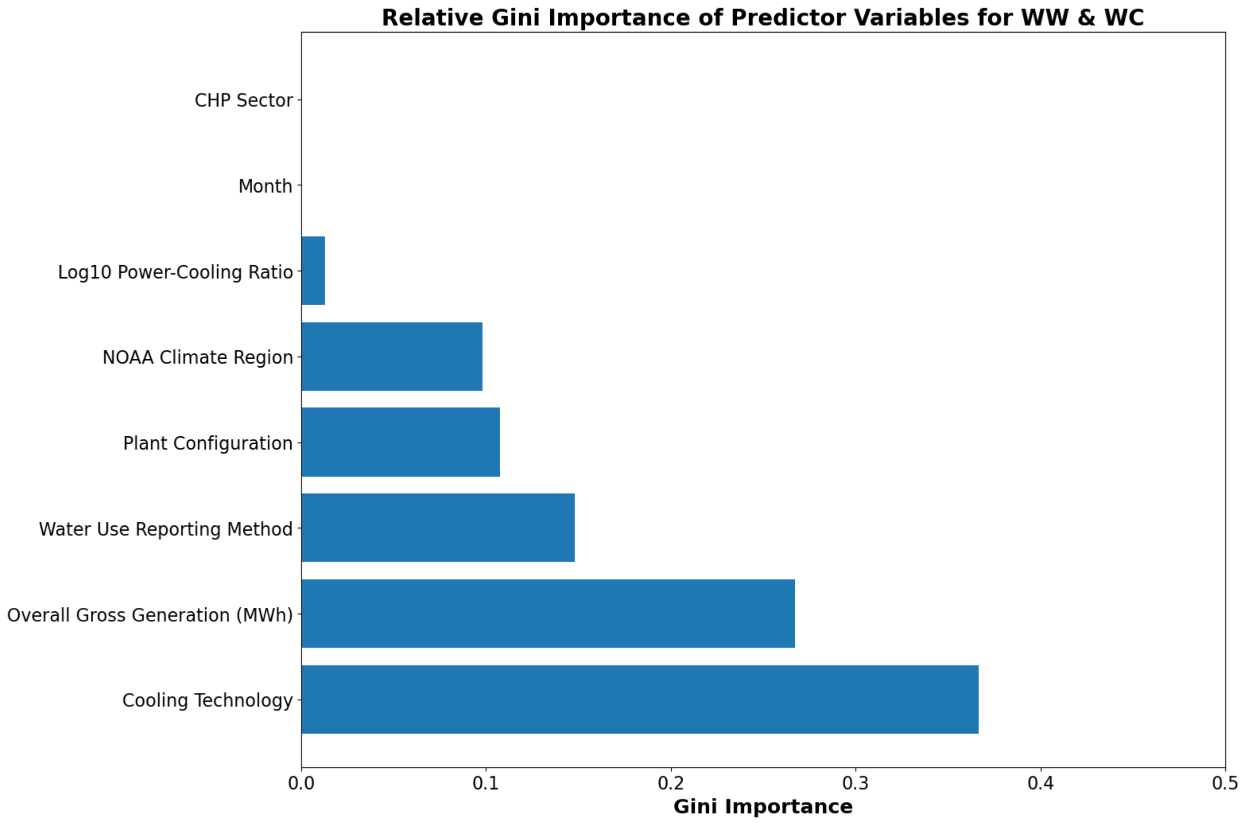

Figure S3 *- Gini importance index of input features for the pruned Multivariate Regression Tree Model (4). Here, the target variables, water withdrawals (WW) and water consumption (WC), are evaluated together.*

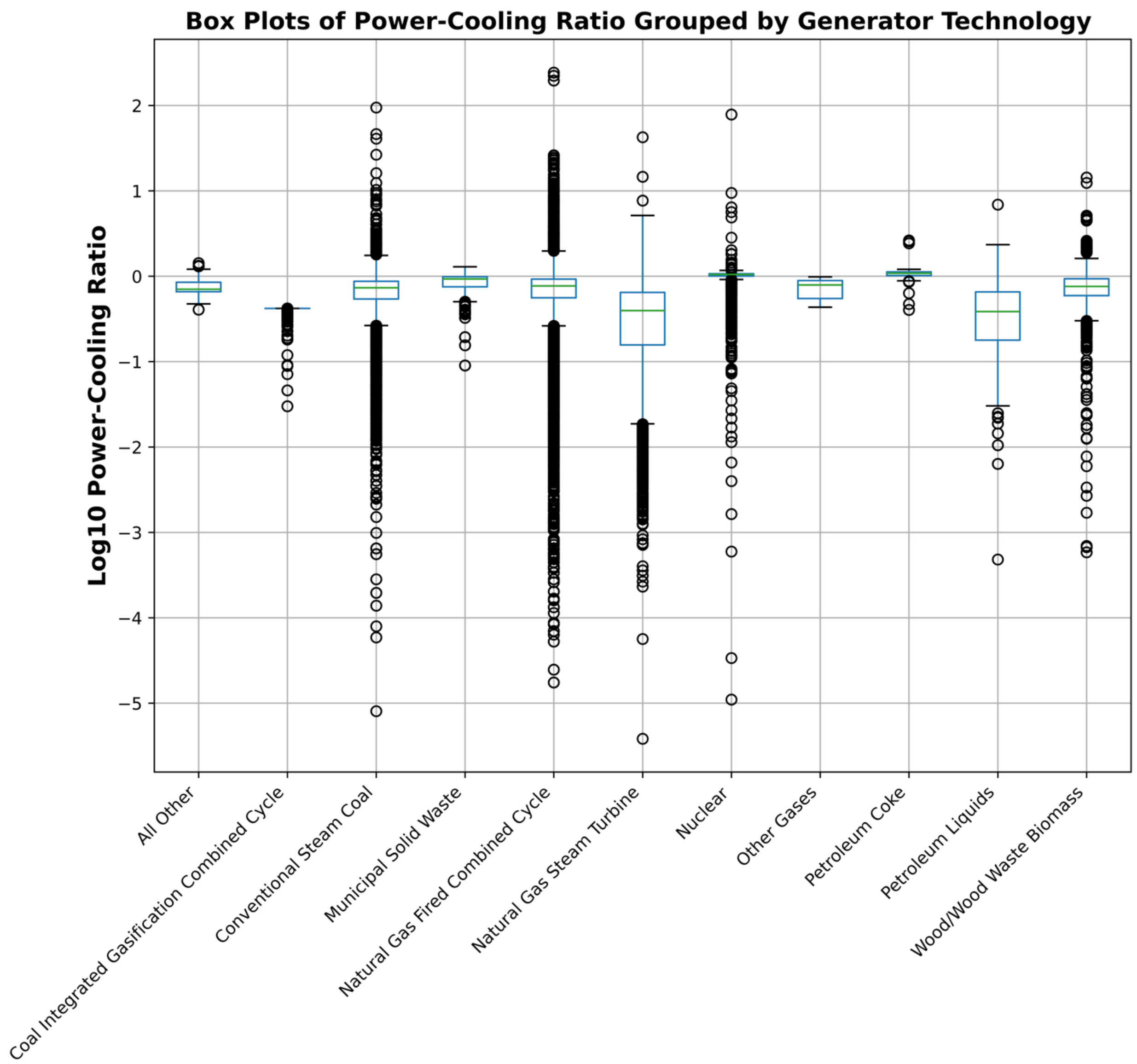

Figure S4 *- Comparison of log10 power-cooling ratio and the generator technology data boxplots across the entire prefiltered dataset. The box spans from the 25*th *percentile (Q1) to the 75*th *percentile (Q3), with the median (50*th *percentile) shown as the vertical line inside the box. The Whiskers extend up to 1.5 times the interquartile range (IQR) beyond Q1 and Q3 and log10 power-cooling ratio values lying outside this range are plotted as outliers.*

Table S1 *- The types of thermoelectric power plant configurations used in this study and the count of each type in the processed EIA dataset. The plant configurations consisted of thermoelectric power plants with 1) one cooling unit, multiple boilers, and multiple generators (1C MB MG), 2) a "Simple" configuration (1C 1B 1G), 3) one cooling unit, one boiler, and multiple generators (1C 1B MG), and 4) one cooling unit, multiple boilers, and one generator (1C MB 1G).*

Thermoelectric Power Plant Figurations	Count
1C, 1B, 1G<br>The 'simple' configuration of 1 cooling system, 1 boiler, and 1 generator	19,853
1C, MB, 1G:<br>Configuration of 1 cooling system, multiple boilers, and 1 generator	2,552
1C, 1B, MG:<br>Configuration of 1 cooling system, 1 boiler, and multiple generators	9,275
1C, MB, MG:<br>Configuration of 1 cooling system, multiple boilers, and multiple generators	125,772
Total	157,452

Table S2 *- NOAA Climate Regions broken down by the states included within each region (6).*

Grouped NOAA Climate Regions	
West	South
California	Arkansas
Nevada	Kansas
	Louisiana
Northwest	Mississippi
Idaho	Oklahoma
Oregon	Texas
Washington	
	Southeast
Southwest	
Arizona	Alabama
Colorado	Florida
New Mexico	Georgia
Utah	North Carolina
	South Carolina
West North Central	Virginia
Montana	
Nebraska	Northeast
North Dakota	Connecticut
South Dakota	Delaware
Wyoming	Maine
	Maryland
East North Central	New Hampshire
Iowa	New Jersey
Michigan	New York
Minnesota	Pennsylvania
Wisconsin	Rhode Island
	Vermont
Central	
Illinois	
Indiana	
Kentucky	
Missouri	
Ohio	
Tennessee	
West Virginia	

Table S3 *- Energy Information Administration Form-923 water use reporting methods aggregated into groups of estimated, measured, permitted, other, and not reported (7). Count refers to numbers of unique plants reporting a given method of water use reporting. A single plant can report multiple different methods of water use reporting through the study period.*

Grouped EIA Reported Water Use Reporting Methods	Count
Measured	238
Measured using a cumulative or continuous flow meter	191
Measured using a streamflow gage or weir	53
Measured using an instantaneous flow meter and pump running time	30
Estimated	266
Estimated based on stated pump capacity and pump running time	100
Consumption calculated as the difference of withdrawal and discharge flows	85
Estimated based on power generation	48
Estimated based on plant design characteristics	29
Estimated based on another flow, such as discharge estimated from measure	27
Consumption estimated from withdrawal amount and loss coefficient	11
Permitted	7
Permitted value, not measured	7
Other	21
Other (provided explanation in Schedule 9)	21
Not Reported	89
NAN values – No reported method of flow measurement	89
Total	663

Table S4 *- Energy Information Administration reported cooling technology categories and an adaptation of EIA abbreviations used (8).*

Cooling Technology Abbreviations	
OT (No CP/C)	Once through without Cooling Pond(s) or Canal(s)
OT (CP)	Once through with Cooling Ponds
R (CP)	Recirculating with Cooling Ponds
R (Forced CP)	Recirculating with Forced Draft Cooling Towers
R (Induced DCT)	Recirculating with Induced Draft Cooling Tower
R (Natural DCT)	Recirculating with Natural Draft Cooling Towers
Hybrid	Hybrid: recirculating with induced draft cooling tower(s) with dry cooling
Dry	Dry cooling system that uses air instead of water to condense steam

Table S5 *- Multivariate Regression Tree full model of predictor features and their definitions.*

Feature	Description
CHP Sector	Different combined heat and power (CHP) sectors describe plants that do and do not produce heat and power in a sequential manner.
Cooling Technology	An equipment system that provides water to the condensers that includes water intakes and outlets; cooling towers; ponds, pumps, and pipes.
Generator Primary Technology	Combination of fuel used and the engine, turbine, water wheel, or similar machine that drives an electric generator, or, for reporting purposes, a device that converts energy to electricity directly.
Log10 Power-Cooling Ratio	The power-cooling ratio with bounds of log10 $\gamma \approx 0$ and log10 $\gamma \approx -1$ that removes infeasible or extreme records to produce a operationally-plausible dataset.
Month	The month of the year in which the plant data was collected and reported by EIA.
NERC Region	The North American Electric Regulatory Council (NERC) regions where the plant is located.
NOAA Climate Region	The allocated NOAA climate region given a plant's spatial coordinates.
Overall Gross Generation	The total generation (megawatt hours) of a plant from steam turbines, single shaft combined cycle units, and combined gas cycle turbines without removing the energy used by the plant itself.
Plant Age	The generator in-service year for the respective power plant.
Plant Configuration	The unique plant configuration between cooling, boiler, and generator systems.
Water Type	The type of water used by the plant: freshwater, reclaimed water, or brackish water.
Water Use Reporting Method	The method used by the plant to report the water used. Grouped as: estimated, measured, permitted, other and not reported.

Table S6 *- Multivariate Regression Tree simplified model of predictor features and their definitions.*

Feature	Description
CHP Sector	Different CHP sectors describe plants that do and do not produce heat and power in a sequential manner.
Cooling Technology	An equipment system that provides water to the condensers that includes water intakes and outlets; cooling towers; ponds, pumps, and pipes.
Log10 Power-Cooling Ratio	The power-cooling ratio with bounds of log10 $\gamma \approx 0$ and log10 $\gamma \approx -1$ that removes infeasible or extreme records to produce a operationally-plausible dataset.
Month	The month of the year in which the plant data was collected and reported by EIA.
NOAA Climate Region	The allocated NOAA climate region given a plant's spatial coordinates.
Overall Gross Generation	The total generation (megawatt hours) of a plant from steam turbines, single shaft combined cycle units, and combined gas cycle turbines without removing the energy used by the plant itself.
Plant Configuration	The unique plant configuration between cooling, boiler, and generator systems.
Water Use Reporting Method	The method used by the plant to report the water used. Grouped as: estimated, measured, permitted, other and not reported.

Table S7 *- A table of all generator and cooling technology combinations from raw data showing how the total counts of unique generator and cooling technology configurations changed when in the prefiltered form, after the Peer & Sanders (9) filter, after the De La Guardia et al. (10) filter, and after this study's power-cooling ratio filter.*

Generator – Cooling Technology Configuration	Prefiltered	Peer & Sanders, 2016	De La Guardia et al., 2022	Log10 Power-cooling Ratio
**All Other**				
R (Induced DCT)	132	0	132	102
**Coal Integrated Gasification Combined Cycle**				
R (CP)	2	0	0	2
R (Induced DCT)	414	66	414	128
**Conventional Steam Coal**				
Dry	24	0	24	24
OT (CP)	307	0	140	300
OT (No CP/C)	5,424	2	4,952	5,106
R (CP)	2,133	879	2,037	1,980
R (Forced DCT)	162	0	140	145
R (Induced DCT)	16,190	215	15,607	14,356
R (Natural DCT)	2,337	345	2,322	2,202
**Municipal Solid Waste**				
R (Induced DCT)	496	0	0	412
**Natural Gas Fired Combined Cycle**				
Hybrid	622	0	622	502
OT (CP)	1,076	588	1,076	1,038
OT (No CP/C)	60	0	60	60
R (CP)	10,434	5,440	10,394	8,380
R (Induced DCT)	102,621	226	98,128	86,366
R (Natural DCT)	60	0	60	60
**Natural Gas Steam Turbine**				
OT (CP)	225	0	225	43
OT (No CP/C)	1,734	131	1,611	1,358
R (CP)	1,720	77	1,617	892
R (Induced DCT)	3,260	16	2,905	2,915
R (Natural DCT)	180	0	141	174
**Nuclear**				
OT (No CP/C)	384	188	169	131
R (CP)	267	226	257	43
R (Induced DCT)	426	399	426	84
R (Natural DCT)	887	811	767	192
**Other Gases**				
OT (No CP/C)	648	0	648	648
**Petroleum Coke**				
R (CP)	2	0	0	2
R (Induced DCT)	112	0	112	19
**Petroleum Liquids**				
OT (No CP/C)	1	0	0	0
R (CP)	82	0	82	58
R (Induced DCT)	452	0	45	379
**Waste/Wood Waste Biomass**				
OT (No CP/C)	200	0	200	190
R (Induced DCT)	4,377	0	2,322	3,614

Table S8 *- A table of all generator and cooling technology combinations from raw data showing how the mean water withdrawal (million gallons) of unique generator and cooling technology configurations changed when in the prefiltered form, after the Peer & Sanders (9) filter, after the De La Guardia et al. (10) filter, and after this study's power-cooling ratio.*

Generator – Cooling Technology Configuration	Prefiltered	Peer & Sanders, 2016	De La Guardia et al., 2022	Log10 Power-cooling Ratio
**All Other**				
R (Induced DCT)	32	N/A	32	24
**Coal Integrated Gasification Combined Cycle**				
R (CP)	7,756	N/A	N/A	7,756
R (Induced DCT)	200	378	200	203
**Conventional Steam Coal**				
Dry	1,490	N/A	1,490	1,490
OT (CP)	5,848	N/A	9,146	5,877
OT (No CP/C)	12,223	14,788	13,019	12,312
R (CP)	18,609	29,493	18,609	18,144
R (Forced DCT)	120	N/A	120	116
R (Induced DCT)	302	490	304	315
R (Natural DCT)	392	469	394	393
**Municipal Solid Waste**				
R (Induced DCT)	25	N/A	N/A	26
**Natural Gas Fired Combined Cycle**				
Hybrid	54	N/A	54	63
OT (CP)	2,954	3,472	2,954	2,956
OT (No CP/C)	19,488	N/A	19,488	19,488
R (CP)	10,569	5,442	10,580	8,696
R (Induced DCT)	77	699	72	74
R (Natural DCT)	46	N/A	46	46
**Natural Gas Steam Turbine**				
OT (CP)	11,006	N/A	11,006	19,292
OT (No CP/C)	10,258	13,973	9,987	10,140
R (CP)	7,532	10,705	7,099	9,372
R (Induced DCT)	353	503	383	312
R (Natural DCT)	72	N/A	84	72
**Nuclear**				
OT (No CP/C)	58,111	34,077	15,022	73,916
R (CP)	59,221	65,432	58,632	47,968
R (Induced DCT)	1,162	1,207	1,162	774
R (Natural DCT)	1,625	1,662	1,189	1,681
**Other Gases**				
OT (No CP/C)	6,448	N/A	6,448	6,448
**Petroleum Coke**				
R (CP)	7,756	N/A	N/A	7,756
R (Induced DCT)	25	N/A	25	16
**Petroleum Liquids**				
OT (No CP/C)	990	N/A	N/A	N/A
R (CP)	42,354	N/A	43,354	45,881
R (Induced DCT)	8	N/A	30	8
**Waste/Wood Waste Biomass**				
OT (No CP/C)	735	N/A	735	739
R (Induced DCT)	499	N/A	330	506

Table S9 *- A table of all generator and cooling technology combinations from raw data showing how the mean water consumption (million gallons) of unique generator and cooling technology configurations changed when in the prefiltered form, after the Peer & Sanders (9) filter, after the De La Guardia et al. (10) filter, and after this study's power-cooling ratio.*

Generator – Cooling Technology Configuration	Prefiltered	Peer & Sanders, 2016	De La Guardia et al., 2022	Log10 Power-cooling Ratio
**All Other**				
R (Induced DCT)	25	N/A	25	21
**Coal Integrated Gasification Combined Cycle**				
R (CP)	1	N/A	N/A	1
R (Induced DCT)	139	261	139	141
**Conventional Steam Coal**				
Dry	1	N/A	1	1
OT (CP)	44	N/A	74	45
OT (No CP/C)	509	233	557	517
R (CP)	299	585	299	297
R (Forced DCT)	120	N/A	120	116
R (Induced DCT)	70	385	67	69
R (Natural DCT)	160	313	161	160
**Municipal Solid Waste**				
R (Induced DCT)	22	N/A	N/A	23
**Natural Gas Fired Combined Cycle**				
Hybrid	35	N/A	35	41
OT (CP)	235	302	235	235
OT (No CP/C)	1	N/A	1	1
R (CP)	2,086	3,823	2,094	2,182
R (Induced DCT)	54	644	54	52
R (Natural DCT)	39	N/A	39	39
**Natural Gas Steam Turbine**				
OT (CP)	21	N/A	21	56
OT (No CP/C)	474	571	488	453
R (CP)	58	413	50	93
R (Induced DCT)	35	420	30	36
R (Natural DCT)	41	N/A	45	41
**Nuclear**				
OT (No CP/C)	597	1,154	699	531
R (CP)	705	821	704	654
R (Induced DCT)	587	616	587	447
R (Natural DCT)	494	530	478	449
**Other Gases**				
OT (No CP/C)	2,206	N/A	2,206	2,206
**Petroleum Coke**				
R (CP)	1	N/A	N/A	1
R (Induced DCT)	13	N/A	13	10
**Petroleum Liquids**				
OT (No CP/C)	990	N/A	N/A	N/A
R (CP)	291	N/A	291	336
R (Induced DCT)	9	N/A	30	10
**Waste/Wood Waste Biomass**				
OT (No CP/C)	332	N/A	332	330
R (Induced DCT)	38	N/A	9	44

Table S10 *- A list of all power plants with reported out-of-bounds operations for 50% or more of the study period (36 months or more) with their Plant ID, name, state and summed mean number of out-of-bounds operations reported.*

Plant ID	Plant Name	State	Summed Mean Number of Out-of-Bounds Months
46	Browns Ferry	AL	38.8
204	Clinton Power Station	IL	38
371	Columbia Generating Station	WA	44
643	Lansing Smith	FL	47
1004	Edwardsport	IN	47.6
1715	Palisades	MI	61
1729	Fermi	MI	42
1925	Prairie Island	MN	59.5
2589	Nine Mile Point Nuclear Station	NY	57
2723	Dan River	NC	38
3452	Lake Hubbard	TX	49.5
3476	Knox Lee	TX	41.5
3490	Graham	TX	57
3491	Handley	TX	55.3
3504	Stryker Creek	TX	41.5
3804	Possum Point	VA	50.6
6008	Palo Verde	AZ	67.3
6015	Harris	NC	60
6020	Perry	OH	62
6073	Victor J Daniel Jr	MS	46.8
6103	TalenEnergy Susquehanna	PA	60.5
6118	PSEG Hope Creek Generating System	NJ	63
6145	Comanche Peak	TX	62.5
6149	Davis Besse	OH	59
6153	Callaway	MO	57
6177	Coronado	AZ	59
6180	Oak Grove	TX	36.5
6462	River Bend	LA	37
7030	Major Oak Power	TX	66
7605	River Bend Gen Plant	WA	45
7722	Watts Bar Nuclear Plant	TN	50
7826	Rowan	NC	42.6
8055	Arkansas Nuclear One	AR	58
10143	Colver Power Project	PA	53
50637	Clearwater Paper IPP Lewiston	ID	37.5
50707	JM Shafer Generating Station	CO	41.6
55126	Milford Power Project	CT	39
55183	Nelson Energy Center	IL	39.75
55210	Afton Generating Station	NM	66
55218	Hinds Energy Facility	MS	41
55220	Attala	MS	39.3
55350	Dresden Energy Facility	PA	45.3
55418	Hot Spring Generating Facility	AR	49.16
55516	Fayette Energy Facility	PA	40
55620	Perryville Power Station	LA	41.3
56671	Longview Power Plant	WV	37

Table S11 *- The counts of each cooling technology pre- and post-PCR filter application to the EIA dataset alongside the percentage of data removed by the PCR filter.*

Cooling Technology	Pre-PCR Filter	Post-PCR Filter	% Removed
Recirculating: Induced Draft	128,481	108,375	15.64%
Recirculating: Cooling Ponds	14,640	11,357	22.42%
Recirculating: Natural Draft	3,464	2,628	24.13%
Recirculating: Forced Draft	162	145	10.49%
Once Through: No Cooling Ponds	8,451	7,492	11.34%
Once Through: With Cooling Ponds	1,608	1,381	14.11%
Hybrid: Dry / Induced Draft	622	502	19.29%
Dry Cooling	24	24	0%

Table S12 *- The counts of each generator primary technology pre- and post-PCR filter application to the EIA dataset alongside the percentage of data removed by the PCR filter.*

Generator Primary Technology	Pre-PCR Filter	Post-PCR Filter	% Removed
Natural Gas Fired Combined Cycle	114,873	96,406	16.07%
Natural Gas Steam Turbine	7,119	5,382	24.39%
Conventional Steam Coal	26,577	24,113	9.27%
Coal Integrated Gasification Combined Cycle	416	130	68.75%
Nuclear	1,964	450	77.08%
Petroleum Liquids	536	437	18.47%
Petroleum Coke	114	21	81.57%
Wood/Waste Biomass	4,577	3,804	17.06%
Municipal Solid Waste	496	412	16.93%
Other Gases	648	648	0%
All Other	132	102	22.72%

Table S13 *- A table of normalized log10 power-cooling ratio out of bounds percentages for each North American Electric Reliability Corporation (NERC) Region (11).*

NERC Regions	Normalized Out of Bounds %
Midwest Reliability Organization (MRO)	12.02%
Northeast Power Coordinating Council (NPCC)	12.7%
ReliabilityFirst Corporation (RFC)	20.10%
SERC Reliability Corporation (SERC)	21.3%
Texas Reliability Entity (TRE)	17.82%
Western Electricity Coordinating Council (WECC)	16.06%

Table S14 *- Comparison of total data records and percentage removed of unique and aggregated water use reporting methods for the raw data vs. power-cooling ratio filtered data.*

Water Use Reporting Method	Total Data Records		
	Total Raw Data	Total Power-Cooling Ratio	% Removed
**Estimated Methods**	**69,496**	**57,432**	17.36%
Estimated based on stated pump capacity and pump running time	16,969	12,930	23.8%
Estimated based on power generation	8,734	7,314	16.26%
Estimated based on plant design characteristics	10,249	8,614	15.95%
Estimated based on another flow, such as discharge estimated from measured withdrawals	4,238	2,928	30.91%
Consumption estimated from withdrawal amount and a loss coefficient	4,534	4,019	11.36%
Consumption calculated as the difference of withdrawal and discharge flows	24,772	21,627	12.52%
**Measured Methods**	**69,343**	**58,893**	15.07%
Measured using a cumulative or continuous flow meter	51,656	43,602	15.59%
Measured using an instantaneous flow meter and pump running time	9,707	8,463	12.82%
Measured using a streamflow gage or weir	7,980	6,828	14.44%
**Other (Provide Explanation in Schedule 9)**	**3,026**	**2,040**	32.58%
**Permitted Value, Not Measured**	**1,113**	**817**	26.59%
**No Reported Method**	**14,474**	**12,723**	12.09%

Table S15 *- Comparison of the percentage of total water withdrawal (left) and percentage of total water consumption (right) volume reported by different water use reporting methods for both the raw data and the filtered data. Note that each column equals 100%.*

**Water Use Reporting Method**	**Percentage of Total Water Withdrawal**		**Percentage of Total Water Consumption**	
	Raw Data	Power-Cooling Ratio	Raw Data	Power-Cooling Ratio
Estimated based on stated pump capacity and pump running time	52.52%	46.3%	17.36%	15.95%
Estimated based on plant design characteristics	0.44%	0.43%	1%	0.9%
Estimated based on another flow, such as discharge estimated from measured withdrawals	0.26%	0.2%	0.42%	0.33%
Estimated based on power generation	5.47%	6.2%	1.96%	1.95%
Consumption estimated from withdrawal amount and a loss coefficient	0.7%	0.87%	5.24%	5.67%
Consumption calculated as the difference of withdrawal and discharge flows	4.73%	4.68%	4.2%	4.21%
Measured using a cumulative or continuous flow meter	2.14%	2.29%	9.16%	8.57%
Measured using an instantaneous flow meter and pump running time	20.51%	25.52%	10.8%	11.66%
Measured using a streamflow gage or weir	8.61%	10.47%	47.3%	48.41%
Other (provide explanation in Schedule 9)	1.61%	1.41%	0.52%	0.37%
Permitted value, not measured	1.86%	0.61%	0.33%	0.25%
No Reported Method	1.15%	1.02%	1.71%	1.73%

Text S4 - ANOVA and Games-Howell Tests of Water Use Reporting Methods

A one-way analysis of variance (ANOVA) is a statistical method used to determine if there are statistically significant differences between the mean of three or more groups (12). It examines the variability between groups to identify if the observed differences are due to actual variation rather than random chance. The ANOVA calculates a F-statistic (Equation S5), which is the ratio between the mean squared difference between groups ($MS_{Between}$) and the mean squared difference within groups (MS_{Within}) and follows a F-distribution.

$$F = \frac{MS_{Between}}{MS_{Within}} \qquad \text{Equation S5}$$

We can then convert the produced F-statistic value to p-value by comparing the F-statistic to the F-distribution for the specific degrees of freedom of the test. The p-value is a statistical metric that aids in understanding if that data provides strong enough evidence to reject the null hypothesis. In this study, the null hypothesis is that there is no difference observed between the water use reporting methods for either water withdrawal or consumption regardless of the power-cooling ratio filter being applied or not. This study uses a p-value threshold of 0.05 to determine statistically significant differences between groups. From Table S15 we observe that for both pre-and post-power cooling ratio application, that aggregated water use reporting method significantly impacts the water withdrawal and consumption values, indicating that each aggregated water use reporting method captures water use data differently.

Table S16 - *Comparison of ANOVA test results between aggregated water use reporting methods and water withdrawal and consumption both pre-and post-power-cooling ratio filter application.*

ANOVA Groups	Pre-PCR Filter	Post-PCR Filter
Water Use Reporting Method and Water Withdrawal	F-statistic: 646.166<br>P-value: <0.00	F-statistic: 358.257<br>P-value: <0.00
Water Use Reporting Method and Water Consumption	F-statistic: 298.437<br>P-value: <0.00	F-statistic: 264.975<br>P-value: <0.00

The Games-Howell post-hoc test is a statistical test used after an ANOVA when we want to compare the means of specific groups to determine which group pairings differ significantly from one another (13). This post-hoc test is useful when the assumption of homogeneity of variances and equal sample sizes are not met, making it a robust post-hoc test compared to other similar methods such as Tukey’s honestly significant difference test. As the aggregated water use reporting methods and their respective volumes of water withdrawal and consumption violate the assumptions of homogeneity of variance and equal sample sizes, the Games-Howell test is the

correct post-hoc test to use. The Games-Howell test uses a modified t-statistic equation (Equation S6) to compare each pair of group means.

$$t_{ij} = \frac{|\bar{X}_i - \bar{X}_j|}{\sqrt{\frac{s_i^2}{n_i} + \frac{s_j^2}{n_j}}} \quad \text{Equation S6}$$

Here $\bar{X}_i$ and $\bar{X}_j$ are the means of groups *i* and *j*, s_i^2 and s_j^2 are the variances between groups *i* and *j*, and n_i and n_j are the sample sizes of groups *i* and *j*. The test calculates the difference between each pair of group means and adjusts for multiple comparisons, controlling the type 1 error rate (false positives where the null hypothesis is incorrectly rejected). To calculate p-values for each pairwise comparisons, the Games-Howell post-hoc test compares the produced modified t-statistic to the t-distribution using an adjusted degrees of freedom based on the Welch-Satterthwaite equation (Equation S7).

$$df = \frac{(\frac{s_i^2}{n_i} + \frac{s_j^2}{n_j})^2}{\frac{(\frac{s_i^2}{n_i})^2}{n_i - 1} + \frac{(\frac{s_j^2}{n_j})^2}{n_j - 1}} \quad \text{Equation S7}$$

Similarly to the ANOVA, the null hypothesis is that there is no significant difference between group pairings and that we use the p-value threshold of 0.05 to determine if there are statistically significant differences between multiple group pairings. From Table S16 we observe that for water withdrawal estimated method volumes are significantly different and greater than measured methods volumes. For water consumption in Table S17 estimated method volumes are significantly different and lower than measure method volumes. Across Table S16 and S17, we see that all water use reporting method group pairings, except for water withdrawal post-PCR filter comparisons of ‘Measured’ vs ‘Other’, ‘Measured’ vs ‘Permitted’, and ‘Other’ vs ‘Permitted’, were statistically significant in their differences from one another, highlighting that water withdrawal and consumption volumes may be overestimated or underestimated depending on the water user reporting methods used.

Table S17 *- Comparison of Games-Howell post-hoc test results between aggregated water use reporting methods and water withdrawal (million gallons) for pre-and post-power-cooling ratio filter application.*

Group 1	Group 2	Pre-PCR Filter		Post-PCR Filter	
		Mean Difference	P-Value	Mean Difference	P-Value
Estimated	Measured	1513.95	<0.00	871.02	<0.00
Estimated	Not Reported	2705.44	<0.00	2208.18	<0.00
Estimated	Other	1256.83	<0.00	766.58	<0.00
Estimated	Permitted	-2394.18	<0.00	647.16	<0.00
Measured	Not Reported	1191.49	<0.00	1337.15	<0.00
Measured	Other	-257.11	<0.00	-104.44	0.27
Measured	Permitted	-3908.13	<0.00	-223.85	0.14
Not Reported	Other	-1448.61	<0.00	-1441.6	<0.00
Not Reported	Permitted	-5099.63	<0.00	-1561.01	<0.00
Other	Permitted	-3651.01	<0.00	-119.41	0.50

Table S18 *- Comparison of Games-Howell post-hoc test results between aggregated water use reporting methods and water consumption (million gallons) for pre-and post-power-cooling ratio filter application.*

Group 1	Group 2	Pre-PCR Filter		Post-PCR Filter	
		Mean Difference	P-Value	Mean Difference	P-Value
Estimated	Measured	-195.47	<0.00	-201.19	<0.00
Estimated	Not Reported	115.61	<0.00	112.67	<0.00
Estimated	Other	92.98	<0.00	99.04	<0.00
Estimated	Permitted	50.29	<0.00	59.8	<0.00
Measured	Not Reported	311.09	<0.00	313.87	<0.00
Measured	Other	288.46	<0.00	300.24	<0.00
Measured	Permitted	245.77	<0.00	261	<0.00
Not Reported	Other	-22.62	<0.00	-13.63	<0.00
Not Reported	Permitted	-65.31	<0.00	-52.86	<0.00
Other	Permitted	-42.69	<0.00	-39.23	<0.00

Supporting Information References

1. De'Ath, G. Multivariate regression trees: a new technique for modeling species–environment relationships. Ecology. **2002**, 83(4), pp.1105-1117.

2. Akaike, H. Information theory and an extension of the maximum likelihood principle. In *Selected papers of hirotugu akaike* (pp. 199-213). New York, NY: Springer New York, **1998**

3. Altmann, A.; Tologi, L.; Sander, O.; Lengauer, T. Permutation importance: a corrected feature importance measure. Bioinformatics. **2010**, 26(10), 1340-1347. DOI: 10.1093/bioinformatics/btq134

4. Hastie, T.; Tibshirani, R.; Friedman, J.H.; Friedman, J.H. The elements of statistical learning: data mining, inference, and prediction; Springer International Publishing: New York City, New York, USA. **2009**.

5. Strobl, C.; Boulesteix, A.L.; Zeileis, A.; Hothorn, T. Bias in random forest variable importance measures: Illustrations, sources and a solution. BMC Bioinformatics. 2007, 8(1), pp.1-21. DOI: 10.1186/1471-2105-8-25

6. National Centers for Environmental Information (NECI); National Oceanic and Atmospheric Administration (NOAA), Geographical Reference Maps: U.S. Climate Regions, **2023**, https://www.ncei.noaa.gov/access/monitoring/reference-maps/us-climate-regions (Accessed on 2023-03-01)

7. Energy Information Administration (EIA), Form EIA-923, **2023**, https://www.eia.gov/electricity/data/eia923/ (Accessed 2023-03-01)

8. Energy Information Administration (EIA), Thermoelectric cooling water data, **2022**, https://www.eia.gov/electricity/data/water/ (Accessed 2023-03-01)

9. Peer, R. A. M.; Sanders, K. T. Characterizing cooling water source and usage patterns across US thermoelectric power plants: a comprehensive assessment of self-reported cooling water data. Environmental Research Letters. **2016**, 11, 124030. DOI: 10.1088/1748-9326/aa51d8

10. De La Guardia, L.; Zhang, Z.; Bai, X. Regional and temporal variability in water use intensity for thermoelectric power plants in the contiguous United States. Journal of Cleaner Production. **2022**, 378, 134604. DOI: 10.1016/j.jclepro.2022.134604

11. North American Electric Reliability Corporation (NERC), ERO Enterprise | Regional Entities, **2024**, https://www.nerc.com/AboutNERC/keyplayers/Pages/default.aspx (Accessed on 2024-02-01)

12. James, G.; Witten, D.; Hastie, T.; Tibshirani, R. An introduction to statistical learning; Springer International Publishing: New York City, New York, USA. **2013**.

13. Games, P.A.; Howell, J.F. Pairwise multiple comparison procedures with unequal n’s and/or variances: a Monte Carlo study. Journal of Educational Statistics. **1976**, 1(2), pp.113-125.
